# The Role of Repetitive Sequences in Repatterning of Major Ribosomal DNA Clusters in Lepidoptera

**DOI:** 10.1093/gbe/evad090

**Published:** 2023-05-24

**Authors:** Martina Dalíková, Irena Provazníková, Jan Provazník, Patrick Grof-Tisza, Adam Pepi, Petr Nguyen

**Affiliations:** Faculty of Science, University of South Bohemia, České Budějovice, Czech Republic; Institute of Entomology, Biology Centre CAS, České Budějovice, Czech Republic; Faculty of Science, University of South Bohemia, České Budějovice, Czech Republic; Institute of Entomology, Biology Centre CAS, České Budějovice, Czech Republic; European Molecular Biology Laboratory, Heidelberg, Germany; Faculty of Science, University of South Bohemia, České Budějovice, Czech Republic; European Molecular Biology Laboratory, Heidelberg, Germany; Institute of Biology, Laboratory of Evolutionary Entomology, University of Neuchâtel, Neuchâtel, Switzerland; Department of Biology, Tufts University; Faculty of Science, University of South Bohemia, České Budějovice, Czech Republic; Institute of Entomology, Biology Centre CAS, České Budějovice, Czech Republic

**Keywords:** satellite, major ribosomal RNA genes, mobile elements, Lepidoptera

## Abstract

Genes for major ribosomal RNAs (rDNA) are present in multiple copies mainly organized in tandem arrays. The number and position of rDNA loci can change dynamically and their repatterning is presumably driven by other repetitive sequences. We explored a peculiar rDNA organization in several representatives of Lepidoptera with either extremely large or numerous rDNA clusters. We combined molecular cytogenetics with analyses of second- and third-generation sequencing data to show that rDNA spreads as a transcription unit and reveal association between rDNA and various repeats. Furthermore, we performed comparative long read analyses among the species with derived rDNA distribution and moths with a single rDNA locus, which is considered ancestral. Our results suggest that satellite arrays, rather than mobile elements, facilitate homology-mediated spread of rDNA via either integration of extrachromosomal rDNA circles or ectopic recombination. The latter arguably better explains preferential spread of rDNA into terminal regions of lepidopteran chromosomes as efficiency of ectopic recombination depends on the proximity of homologous sequences to telomeres.

**Significance**

Clusters of genes for major ribosomal RNAs (rDNA) can move throughout the genome. Yet, their mobility is not fully understood. In moths and butterflies, analyses of both short and long reads revealed the presence of various repetitive sequences in rDNA intergenic spacers. Comparison between species with ancestral and derived distribution of rDNA suggests satellite DNA is instrumental for homology-mediated spread of rDNA.

## Introduction

Ribosomal RNAs (rDNA) have a central role in ribosome functions in protein synthesis and thus are a cornerstone for life as we know it (Noller et al. 2017). They are shared by all eukaryotes and have been considered the oldest repetitive fraction (Symonová 2019) as their genes are present in multiple copies mainly organized in tandem arrays. The genes for major rDNA, that is, 18S, 5.8S, and 28S in animals, form a transcription unit, in which internal transcribed spacers (ITS 1 and 2) separate individual genes. In eukaryotic genomes (Prokopowich et al. 2003), there are hundreds or even thousands of rDNA units separated by intergenic spacers (IGS) (Long and Dawid 1980).

Sequences of rRNA genes and their transcribed spacers have been used in taxonomy for species identification (Wu et al. 2015) or to reconstruct phylogenetic relationships (Fiore-Donno et al. 2012). Moreover, thanks to their cluster organization, the rDNA can be easily detected on chromosomes by fluorescence in situ hybridization (FISH), which makes it an important marker in cytogenetic studies (Nguyen et al. 2010; Palacios-Gimenez et al. 2013; Ferretti et al. 2019; Provazníková et al. 2021). The rDNA clusters can be present on autosomes, sex chromosomes, or even supernumerary chromosomes, that is, B chromosomes (Poletto et al. 2010; Cabral-de-Mello et al. 2011; Silva et al. 2014; García et al. 2017; Zrzavá et al. 2018; Provazníková et al. 2021). While most animal species have only one rDNA locus, up to tens of loci were reported in some extreme cases (Eickbush and Eickbush 2007; Sochorová et al. 2018 and references therein). The active loci are also called the nucleolar organizer regions (NORs) (Ingle et al. 1975; Kobayashi 2011), as transcription of major rDNA genes and processing of primary transcripts give rise to a subnuclear compartment known as a nucleolus (reviewed in Eickbush and Eickbush 2007). In general, changes in distribution of rDNA genes are dynamic and rDNA was thus compared with mobile elements (MEs), which, in turn, have been considered an important driver in rDNA repatterning (Cabral-de-Mello et al. 2011; Scacchetti et al. 2012; Elliott et al. 2013; de Sene et al. 2015; Ferretti et al. 2019).

The order Lepidoptera with its 160,000 species of moths and butterflies represents one of the largest insect radiations (Van Nieukerken et al. 2011). Their rich species and ecological diversity contrast with their conserved genome architecture with the ancestral and the most common chromosome number being *n* = 31 (Robinson 1971; Van’t Hof et al. 2013; Ahola et al. 2014). Detailed analyses of advanced ditrysian species, such as the peppered moth (*Biston betularia*, Geometridae, *n* = 31; Van’t Hof et al. 2013), the Glanville fritillary (*Melitaea cinxia*, Nymphalidae, *n* = 31; Ahola et al. 2014), and the tobacco cutworm (*Spodoptera litura*, Noctuidae, *n* = 31; Cheng et al. 2017), showed highly conserved synteny and order of genes between homoeologous chromosomes (Van’t Hof et al. 2013; Ahola et al. 2014). A typical lepidopteran mitotic complement consists of small dot-shaped chromosomes (Prins and Saitoh 2003; Mediouni et al. 2004; Fuková et al. 2005), which lack localized centromere, that is, they are holokinetic (Wolf et al. 1997). Moreover, traditional banding techniques failed to differentiate individual chromosomes, which has made the classic cytogenetic research in Lepidoptera rather challenging (Bedo 1984) and limited it, for a long time, only to chromosome counting (Robinson 1971; Lukhtanov 2015). However, FISH with probes derived from whole genomes, microdissected chromosomes, or bacterial artificial chromosomes provided great insight into evolution of lepidopteran karyotypes (Yasukochi et al. 2011; Van’t Hof et al. 2013), sex chromosomes (Vítková et al. 2007; Šíchová et al. 2015; Dalíková et al. 2017a; Carabajal Paladino et al. 2019), repetitive sequences (Šíchová et al. 2013, 2015), and gene families such as major rDNA (Nguyen et al. 2010; Provazníková et al. 2021).

Number and localization of rDNA loci were determined using FISH with the 18S rRNA probe in about 30 species sampled across 14 lepidopteran superfamilies (Fuková et al. 2005; Nguyen et al. 2010; Vershinina et al. 2015; Šíchová et al. 2013, 2015, 2016; Zrzavá et al. 2018; Provazníková et al. 2021). The results implied that one terminal rDNA cluster per haploid genome is probably the ancestral state as it was found across all Lepidoptera (supplementary fig. S1, Supplementary Material online). In some ditrysian families, such as Noctuidae and Erebidae (supplementary fig. S1, Supplementary Material online), the rDNA cluster moved to interstitial position, which was conserved in all studied species (Nguyen et al. 2010; Provazníková et al. 2021). When multiple clusters are present, they are located terminally in majority of species. Higher numbers of rDNA clusters were observed in representatives of the families Psychidae (3–4 clusters per haploid genome) and Nymphalidae (7–11 clusters per haploid genome), *B. betularia* (3 clusters per haploid genome, Geometridae), and *Hyalophora cecropia* (3 clusters, Saturniidae), all having the ancestral haploid chromosome number *n* = 31 (supplementary fig. S1, Supplementary Material online). Thus, spread of rDNA clusters is not clearly associated with large-scale chromosome rearrangements such as chromosome fissions or fusions. Unusual distribution of rDNA was observed in the ghost moth, *Hepialus humuli* (Hepialidae), and the horse chestnut leaf miner, *Cameraria ohridella* (Gracillariidae), in which signal of the 18S rDNA probe covered about one-half and one-fourth of a single NOR–bearing chromosome pair, respectively (Provazníková et al. 2021) (supplementary fig. S1, Supplementary Material online). It was proposed that the dynamic rDNA repatterning is due to ectopic recombination, that is, recombination between nonhomologous regions mediated by ubiquitous repetitive sequences (Nguyen et al. 2010). However, the hypothesis is yet to be tested.

In this study, we decided to test for a role of repetitive sequences in repatterning of major ribosomal genes in *H. humuli* and *C. ohridella* with extremely large rDNA cluster and nymphalids *Aglais urticae* and *Inachis io* with seven and eleven loci per haploid genome, respectively. We performed FISH with probes for 18S and 28S rDNA to test whether genes for major rRNAs spread individually or as a transcription unit (cf. Symonová et al. 2013; Ferretti et al. 2019). Further, we sequenced genomes of all four species and analyzed repetitive sequences and their colocalization with rDNA using the RepeatExplorer pipeline (Novák et al. 2013, 2010). We estimated a portion of rDNA units associated with identified repeats by analyses of coverage. The colocalization of several repetitive sequences with rDNA was verified by FISH and in *H. humuli* and the nymphalids also by analysis of long reads. The long read analysis was further performed also in *Phymatopus californicus* (Hepialidae) to compare it with the peculiar *H. humuli* rDNA organization. Finally, we compared these species with highly derived rDNA distribution and three moths, namely, *Lymantria dispar* (Erebidae), *Spodoptera frugiperda* (Noctuidae), and *Plutella xylostella* (Plutellidae), with a single, presumably ancestral, rDNA locus. Our work shows that combining molecular cytogenetic techniques with next-generation sequencing technologies represents a powerful tool to study evolution of genome architecture in Lepidoptera.

## Results

### FISH with 18S and 28S rDNA Probes

**Fig. 1.**
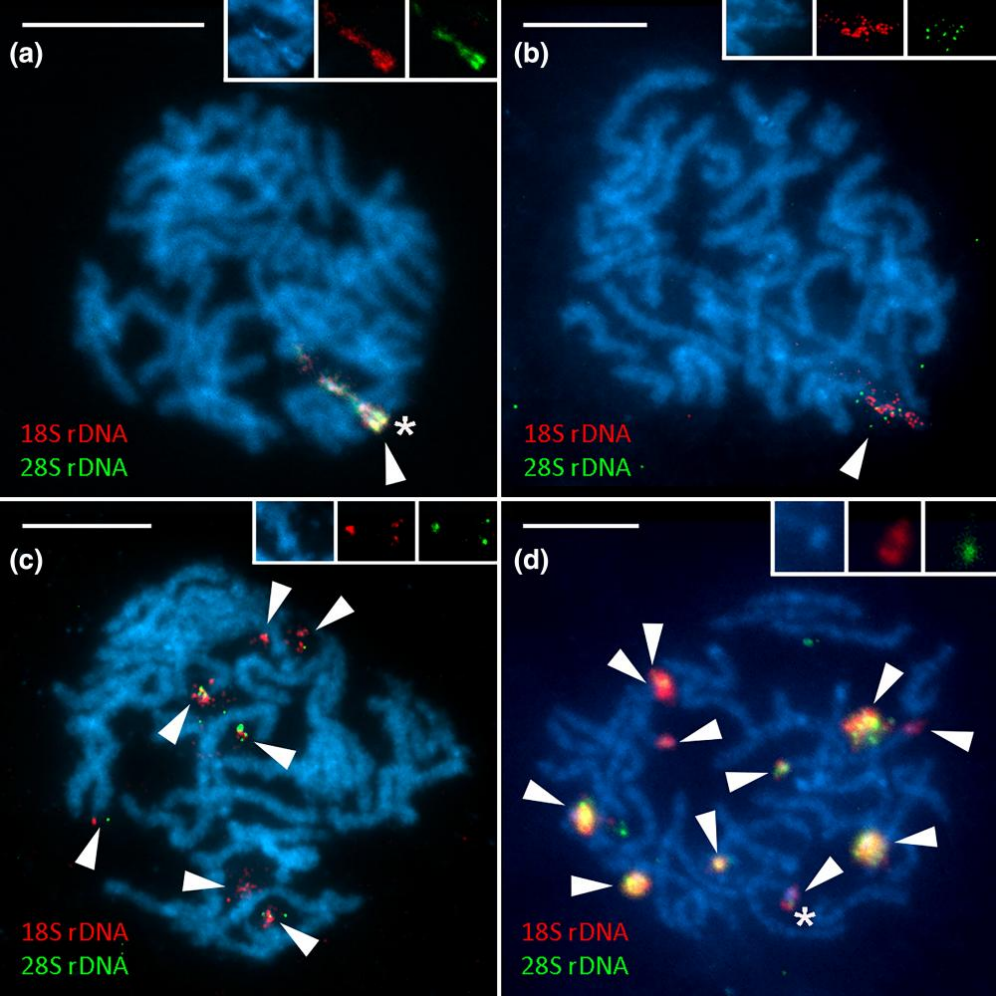
Major rDNA clusters detected by FISH on pachytene nuclei of studied species. The 18*S* rDNA probe (red) and 28*S* rDNA probe (green) chromosomes are counterstained with DAPI (blue). Major rDNA clusters indicated by arrowheads. (*a*) Male pachytene nucleus of *H. humuli* (Hepialidae) with detail of the major rDNA cluster colocalizing with heterochromatin blocks in the inset, (*b*) male pachytene nucleus of *C. ohridella* (Gracillariidae), (*c*) male pachytene nucleus of *A. urticae* (Nymphalidae), and (*d*) female pachytene nucleus of *I. io* (Nymphalidae) with detail of one of the major rDNA clusters colocalizing with a small heterochromatin block in the inset. Asterisks indicate clusters colocalizing with DAPI-positive heterochromatin. Scale 10 *µ*m.

To examine the organization of the rDNA clusters in genomes of four studied species, namely, *H. humuli*, *C. ohridella*, *A. urticae*, *and I. io*, simultaneous FISH with both 18S and 28S rDNA probes was carried out. Hybridization patterns of the 18S rDNA probe of all four species correspond to previous results (Nguyen et al. 2010; Provazníková et al. 2021). Moreover, the 28S rDNA probe colocalized with the 18S rDNA probe in all cases which suggests that the observed patterns of rDNA distribution are due to the spread of the whole rDNA unit. The two signals did not consistently overlap, which is probably artefact caused by enzymatic detection producing signals of varying intensity (cf. Rego and Marec 2003) and nonuniform emission spectrum of mercury lamp causing weaker excitation of green fluorochrome. One large major rDNA cluster covering a large portion of one chromosomal bivalent was observed in pachytene nuclei of *H. humuli* (fig. 1*a*) and *C. ohridella* (fig. 1*b*). Additionally, a strong DAPI-positive heterochromatin block colocalized with a major rDNA cluster in *H. humuli* (fig. 1*a* detail). In pachytene nuclei of *A. urticae* and *I. io*, multiple rDNA clusters were observed as previously described. Seven small terminal clusters in pachytene nucleus of *A. urticae* did not colocalize with any heterochromatin blocks (fig. 1*c*), whereas in pachytene nucleus of *I. io*, 11 terminal signals of various sizes were detected and 6 of them colocalizing with small DAPI-positive blocks (example in fig. 1*d* detail). Numerous chromosomal bivalents bearing small terminal heterochromatin blocks seem to be a typical feature for the *I. io* karyotype.

### Repeat Explorer Analysis

To identify repeats associated with 45S rDNA, we performed a Repeat Explorer (RE) analysis in all four studied species. Repetitive sequences with frequent colocalization in the genome can be identified through RE analysis as clusters connected by pair-end reads forming the so-called superclusters. In *C. ohridella*, major rDNA genes were split into two clusters (supplementary fig. S2*a*, Supplementary Material online). Surprisingly, these clusters had no connection to each other or to any other identified repeat. As a result, we were not able to reconstruct the rDNA consensus sequence in this species as the individual contigs from both clusters did not overlap. The estimation of the genome proportion formed by major rDNA in this species is about 0.04% (supplementary Table S1, Supplementary Material online). In *I. io*, clusters annotated as 45S rDNA were part of supercluster 11. This supercluster was formed by three clusters which were annotated as 28S and LINE R2 element (cluster 27), 18S and 5.8S (cluster 35), and putative satellite (IiSat, cluster 37) with a predicted monomer length of 157 bp (supplementary Table S1 and fig. S2*b*, Supplementary Material online). As cluster 27 was formed by both the 28S rRNA gene and LINE R2 elements (IiR2), the genome proportion of major rDNA cannot be determined with certainty from the RE results alone; but these genes could comprise 0.12–0.29% of the *I. io* genome (supplementary Table S1, Supplementary Material online). In *A. urticae*, genes for major rDNA were also divided into several clusters. Three clusters 17, 21, and 29 annotated as 45S rDNA formed one supercluster 8 and were not connected to any other repeat by 10 or more shared pair-end reads (supplementary Table S1 and fig. S2*c*, Supplementary Material online). However, after further inspection, one contig corresponding to cluster 29 contained tandemly repeated sequence suggesting that a satellite repeat (AuSat) with monomer approx. 400 bp is part of this cluster. Based on the RE estimate, the major rDNA clusters formed 0.59% of the *A. urticae* genome. In *H. humuli*, major rDNA genes represented 0.17% of the genome and were all comprised in cluster 53 which was part of supercluster 24 together with cluster 67 annotated as ME from the Ty3/Gypsy group (Hh Ty3/gypsyA; supplementary table S1, Supplementary Material online, supplementary fig. S2*d*, Supplementary Material online).

### FISH with 18S rDNA and Probes Derived from MEs

**Fig. 2.**
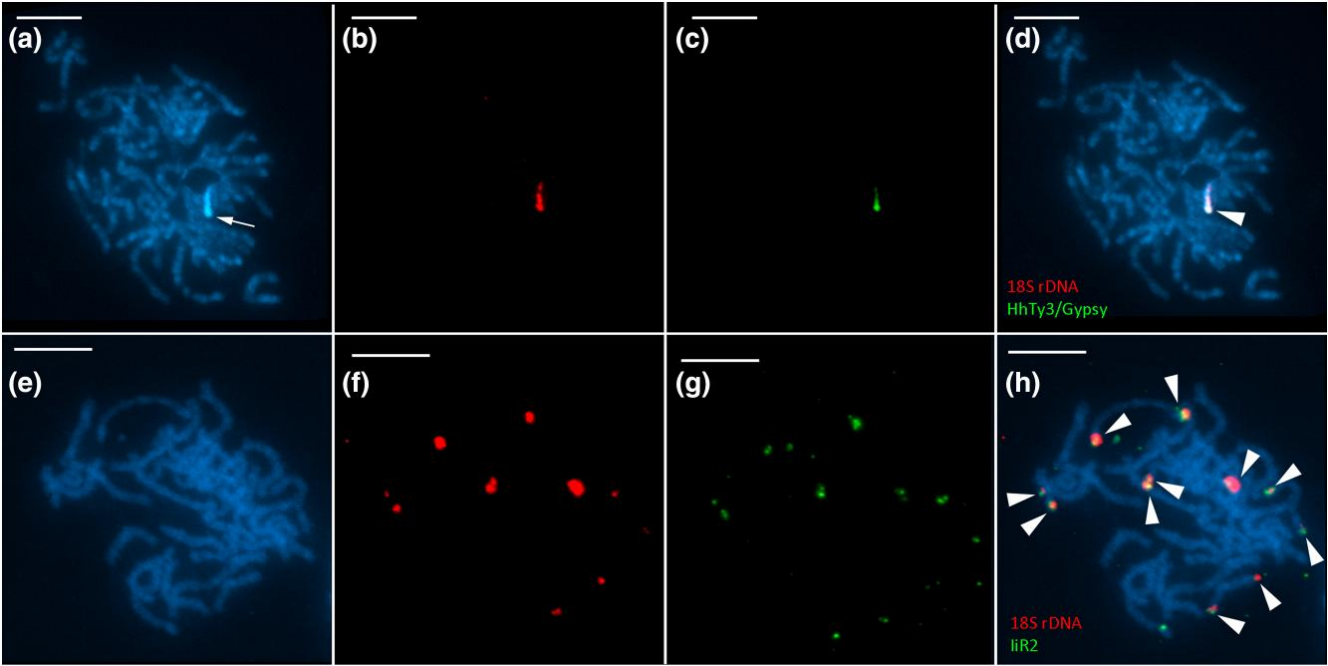
Colocalization of major rDNA and ME sequences of interest as detected by FISH with tyramide signal amplification on pachytene nuclei of studied species. 18*S* rDNA probe, in red, (*b*, *d*, *f*, and *h*) and HhTy3/GypsyA (*c*, *d*) and IiR2 probes (*g*, *h*), in green, chromosomes are counterstained with DAPI (blue; *a*, *d*, *e*, and *h*). Hybridization signals indicated by arrowhead. (*a*–*d*) Male pachytene nucleus of *H. humuli* (Hepialidae) and (*e*–*h*) female pachytene nucleus of *I. io* (Nymphalidae). The arrow indicates DAPI-positive heterochromatin. Scale 10 *µ*m.

To verify the results obtained from RE analysis, we mapped the ME probe together with the 18S probe on chromosome preparations of *H. humuli* and *I. io* by double TSA FISH. In *H. humuli*, both the 18S rDNA probe and the pooled Hh Ty3/GypsyA *RT*, *INT*, and *RH* probes hybridized to the major rDNA cluster region (fig. 2*d*) and thus confirmed association between Hh Ty3/GypsyA and the major rDNA. Similar pattern was observed in *I. io*, in which both the 18S rDNA probe and the probe for IiR2 *RT* hybridized to all 11 clusters of major rDNA in pachytene nuclei (fig. 2 h), thus proving an association between rDNA and the IiR2 ME. Hybridization of the IiSat probe did not provide any clear signal which was probably caused by the high AT content of the probe and both length and sequence variation between satellite monomers. Additionally, to investigate whether IiR2 is also present in the genome of closely related *A. urticae*, we hybridized the 18S rDNA probe and the IiR2 probe to pachytene nuclei of *A. urticae*. However, no clear hybridization signal was observed (results not shown).

### Long Read Analysis

**Fig. 3.**
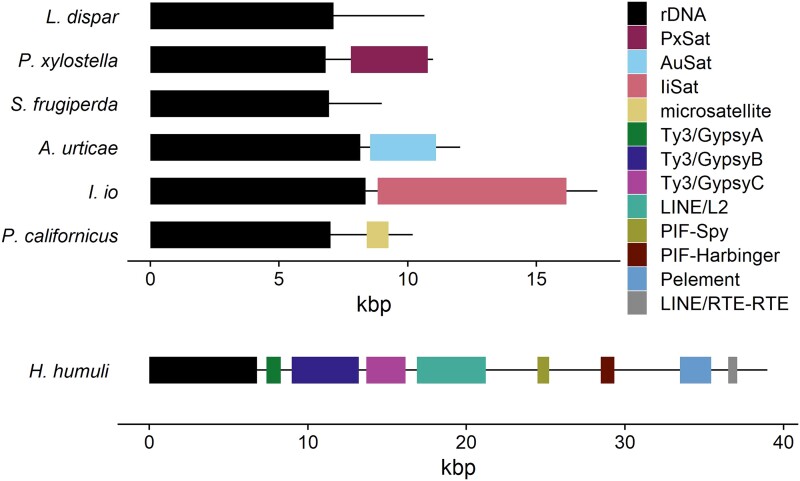
Schematic representation of the most represented rDNA unit based on long read analysis. Black line represents the length of rDNA unit and colored bars represent rDNA genes including intergenic transcribed spacers (ITS1 and ITS2) and the repetitive elements found in intergenic spacers. Positions and lengths of individual segments are summarized in supplementary table S2, Supplementary Material online.

Long read analysis was used to further verify connection between rDNA and repeats revealed by RE and FISH results. Output of the *H. humuli* sequencing run was poor both in overall yield and read length. After default quality filtering, which was part of the base calling process, we obtained 4 Gb in reads with a N50 length of 6 kb. Due to low coverage of obtained Nanopore data, we were able to analyze only 567 reads longer than 15 kb with mean quality (*Q*) > 10 containing major rRNA genes in *H. humuli*. Most of these reads contained nonfunctional short copies of three Ty3/Gypsy elements, two LINE elements (from L2 and RTE-RTE groups), two PIF elements (Harbinger and Spy group), and a *P* element in the IGSs. The length of these MEs ranged from 588 bp (fragment of RTE-RTE element) to 4.3 kb (L2 element) (fig. 3 and supplementary Tab. S2, Supplementary Material online). The organization and length of the IGSs were highly conserved. Only 18 reads bearing rDNA did not contain any of the mentioned MEs, and around 12% of the reads exhibited some irregularities in the observed pattern (supplementary fig. S3, Supplementary Material online). All reads containing rDNA were used to assemble rDNA unit including IGS. The resulting Flye assembly contained a single circular contig 77,920 bp long corresponding to two complete rDNA units differing in nine nucleotides and four indels with a cumulative length of 6 bp. The longer rDNA unit is visualized in figure 3. Although only one of the observed Ty3/Gypsy elements, Hh Ty3/gypsyA, was detected by the RE analysis as a part of the supercluster-containing rDNA, upon careful examination of RE results, all the IGS repeats were connected by shared pair-end reads. Yet, these did not suffice to bind rDNA and all associated MEs in one supercluster (supplementary Table S1, Supplementary Material online).

To test for the presence of such complex IGS in other representative of the family Hepialidae, we have analyzed also Nanopore reads of *P. californicus*. After base calling with default quality filtration, we obtained 40 Gb of data with read length N50 of 22 kb. Total of 596 reads bearing major rDNA passed the filtering for length > 15 kb and *Q* > 10. Yet, in this case, the IGSs were much smaller and contained only ca. 800 bp long microsatellite region (supplementary fig. S4, Supplementary Material online) consisting of sequence complementary to insect telomeric repeat TTAGG and 231 bp long region of the TTATG microsatellite. The Flye assembly yielded a single 30,616 bp long circular contig. However, this contig corresponded to three complete major rDNA units which differed in length of the microsatellite region by up to four TTAGG repeats. The major rDNA unit in *P. californicus* is thus about 10 kb long including the IGS region. The consensus of the complete rDNA unit is visualized in figure 3.

Due to the high coverage of available HiFi PacBio reads of *I. io*, we were able to analyze 3,276 reads containing major rDNA longer than 15 kb (supplementary fig. S5, Supplementary Material online). Of these, 2,625 contained at least 200 bp of the satellite recovered by the RE analysis in their IGS. However, the individual major rDNA units differed in length of this satellite array (supplementary Table S3, Supplementary Material online). In 946 reads, the R2 element was inserted in major rDNA genes. Surprisingly, some of these insertions were not limited to the 28S rRNA gene, suggesting ongoing degeneration of rDNA units via R2 insertions in *I. io*. The attempt to assemble the most prevalent variant of complete rDNA unit in *I. io* failed as all assemblies contained more than 300 contigs of variable length, both linear and circular. However, most of the contigs had very low coverage. Moreover, only 27 contigs contained more than 200 bp of any rRNA gene. Out of all obtained contigs, only three had mean coverage over 10% of used PacBio reads and their length varied from approx. 15.8–33 kb. These three contigs all contained at least some of the major rRNA genes either with or without R2 element insertion and the satellite array with variable lengths from 4.5 to 17 kb. This further emphasizes the variability in IGSs in this species. The rDNA unit from the contig with the highest coverage is visualized in figure 3.

In *A. urticae*, the available HiFi PacBio data contained 2,921 reads > 15 kb bearing major rDNA. The long read analysis confirmed the overall lack of MEs associated with rDNA (supplementary fig. S6, Supplementary Material online) as only 179 reads contained either LINE or Ty3/Gypsy element adjacent to major rRNA genes, including R2 element inserted into 28S rDNA observed in 49 reads. Despite the absence of MEs in IGS of *A. urticae*, this region seems to vary in length between major rDNA units (supplementary fig. S6, Supplementary Material online). The variation of IGS was reflected also in assembly results as any assembly produced by Flye contained over 70 linear contigs in the length ranging 19–69 kb. However, only six contigs contained more than 200 bp of major rDNA and only one of these contigs had mean coverage over 10% of input reads. This contig is 31 kb long and contains two complete rDNA units including IGS regions. One unit contains R2 insertion in the 28S gene, and based on dot plot, both IGSs contain approx. 2 kb of satellite (AuSat) array. Both AuSat IGS satellite arrays contain six monomers with a variable length from 252 to 408 bp with the last monomer being the shortest one. The complete unit consensus is visualized in figure 3. This satellite was present in most major rDNA units as it was found in 2,805 reads containing major rDNA (supplementary fig. S6, Supplementary Material online). After further inspection of previous RE results in this species, a sequence homologous to the satellite was found among contigs belonging to cluster 29 (supplementary Table S1, Supplementary Material online) which also contained a part of the 28S rRNA gene. Interestingly, our RE results did not contain any cluster with sequence homologous to the AuR2 element found in long reads. Considering that samples for RE were sampled from the Czech *A. urticae* population while specimen sequenced by PacBio originated from the Great Britain, the R2 insertion into rDNA units may represent interpopulation variation in this species.

To test if the variable and/or long IGSs are connected with atypical rDNA genomic organization, we performed long read analysis also in species with one major rDNA locus per haploid genome which is supposedly ancestral in Lepidoptera (Nguyen et al. 2010; Provazníková et al. 2021), namely, in *P. xylostella*, *S. frugiperda*, and *L. dispar*. In *P. xylostella*, we analyzed 1,437 HiFi PacBio reads containing major rDNA with a length of at least 15 kb. The rDNA was rarely associated with any ME; however, 84 reads contained either LINE or Ty3/Gypsy element including R1, the latter being inserted in 50 reads (supplementary fig. S7, Supplementary Material online). Moreover, the IGS contained the satellite (PxSat) region (supplementary fig. S7, Supplementary Material online). This array consisted of several monomers with a slightly variable length between 248 and 258 bp and even some incomplete monomers being only 91 bp long. At least 200 bp of this satellite array was found in 1,397 reads containing major rDNA. The Flye assembly of all filtered rDNA bearing PacBio reads yielded one scaffold and four contigs with lengths ranging from 2 to 39 kb. The scaffold had the highest coverage and was 14 kb long. It contained one complete and one partial unit. The two largest contigs contained rDNA units associated with various MEs (supplementary fig. S7, Supplementary Material online), and one of them also contained insect telomeric repeat at the end suggesting the terminal rDNA loci in *P. xylostella* is adjacent to the telomere. The consensus of complete rDNA unit is visualized in figure 3.

In *L. dispar*, 242 quality-filtered PacBio reads contained major rDNA. Surprisingly, major rDNA in this species was associated with two different MEs from the LINE R1 group specific for rDNA (supplementary fig. S8, Supplementary Material online). 51 analyzed reads contained at least one of those elements, and eight reads contained both transposons. Flye assembly contained two linear contigs 11 kb and 9.4 kb long with the latter having approx. 10× higher coverage. Neither of these contigs contained complete major rDNA unit, the longer contig contained both R1 elements and the shorter one incomplete major rDNA unit with IGS but only a partial 28S rRNA gene.

In *S. frugiperda*, we analyzed 115 quality and length-filtered PacBio reads containing major rDNA. There were no MEs or satellite sequences observed in these reads (supplementary fig. S9, Supplementary Material online). Flye assembly consisted of only one circular contig 17.9 kb long which contained two identical complete major rDNA units.

Satellite DNA arrays contained in IGSs of *P. xylostella* representing the ancestral rDNA distribution and both nymphalid species with multiple rDNA clusters seemed to vary in length. Thus, we further characterized these satellite arrays. All three species have similar most represented array length in the PacBio reads, as the medians are ranging from 1.92 to 2.21 kb (supplementary Table S3 and fig. S10, Supplementary Material online). However, they differ greatly in maximal observed length, which was 5.83 kb in *P. xylostella* but over 15 kb in both nymphalid species (supplementary Table S3 and fig. S10, Supplementary Material online). Similar differences can be seen in the satellite array length in the rDNA assemblies. In nymphalid species, we obtained a much higher number of contigs with variable satellite length ranging from less than 1 kb to 2.23 kb in *A. urticae* and over 17 kb in *I. io*. (supplementary table S3, Supplementary Material online). These results suggest higher variation in length of IGS satellite arrays in *A. urticae* and *I. io* compared with *P. xylostella* (supplementary Table S3 and fig. S10, Supplementary Material online). Moreover, the three species differed in the presence of PacBio reads containing satellite sequence without any part of the rDNA unit. While we found no such reads in *P. xylostella*, 11 and 30 reads were found in *A. urticae* and *I. io* PacBio data, respectively. As the lengths of PacBio reads bearing only satellite in both nymphalid species exceed the lengths of observed satellite arrays in rDNA assemblies (supplementary Table S3, Supplementary Material online), these reads either come from very large IGS satellite arrays or they may represent satellite arrays outside the rDNA cluster. The latter is supported by the recently published *A. urticae* genome (Bishop et al. 2021), which, however, contains only six terminal rDNA loci. AuSat is found both within the IGS region and right outside two NORs (supplementary fig. S11, Supplementary Material online).

To compare the intragenomic variability of rDNA genes and ITS in *P. xylostella* and both nymphalid species, we performed clustering analysis and compared average pairwise identity and percentage of identical sites. In the clustering analysis, we used two thresholds to separate individual rDNA gene regions (18S, ITS1, 5.8S, ITS2, and 28S) into clusters. In both analyses, *P. xylostella* exhibited a lower number of clusters compared with *A. urticae* and *I. io* (supplementary fig. S12, Supplementary Material online). With an 80% identity threshold, *P. xylostella* sequences split into 8 clusters compared with 23 in each nymphalid species, whereas, 95% identity analysis produced 18 clusters in *P. xylostella* and over 60 clusters in *A. urticae* and *I. io*. The 95% identity clustering also suggests that even in species with a single cluster, the sequences are not completely homogenized as even in *P. xylostella*, several clusters contained genes from a similar number of rDNA units (supplementary fig. S12*b*, Supplementary Material online). Although the average pairwise identity is similar for all rDNA genes and both ITS among all species, *P. xylostella* has a higher percentage of identical sites for all parts of transcribed regions of the rDNA unit compared with either nymphalid species, with the only exception being ITS1 (supplementary Table S4, Supplementary Material online).

### Coverage Analysis

**Fig. 4.**
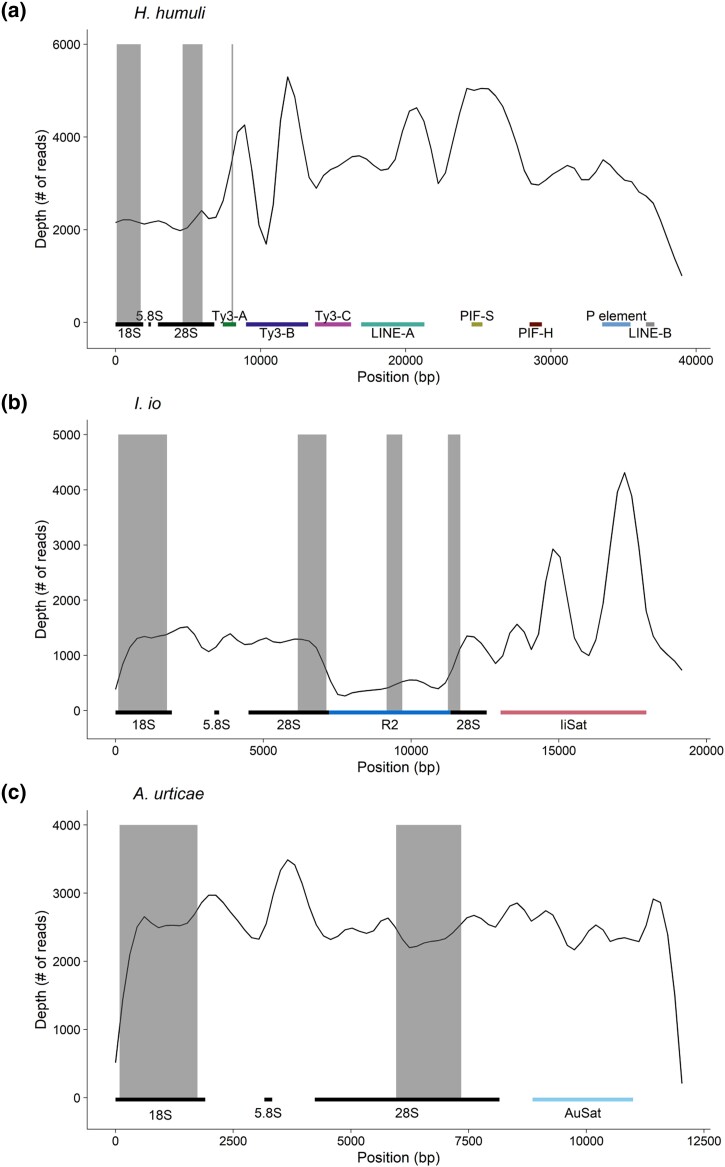
Coverage plot of rDNA units in *H. humuli* (*a*), *I. io* (*b*), and *A. urticae* (*c*). Smoothed (LOESS) counts of aligned sequencing reads for each nucleotide position of the major rDNA cluster. Colored bars on the bottom represent regions of repetitive elements and position of rDNA genes. The gray vertical bars represent position of the successfully hybridized probes. Ty3, Ty3/Gypsy; LINE-A, LINE/L2; PIF-S, PIF-Spy; PIF-H, PIF-Harbinger; LINE-B, LINE/RTE-RTE.

Paired-end reads produced by Illumina sequencing provided us with sufficient coverage to compare per base abundance of reads aligned to the consensus sequences of the whole rDNA unit of *H. humuli* obtained via long reads analysis (see above) and the most represented complete rDNA unit of *I. io* and *A. urticae* from rDNA–assembled contigs (see above). In case of *H. humuli* (fig. 4*a* and supplementary table S5, Supplementary Material online), the repetitive elements associated with major rDNA are most likely present elsewhere in the genome as we observed a uniform coverage of the rDNA genes and varying but higher coverage of the intergenic MEs. In *I. io*, we observed that the R2 element is only present in roughly one-third of the copies of the 28S rRNA gene (fig. 4*b* and supplementary table S5, Supplementary Material online). The coverage of the IiSat region was approx. two times larger compared with rRNA genes (fig. 4*b* and supplementary Tab. S5, Supplementary Material online), which similarly to the results obtained from PacBio reads suggest the IiSat presence outside of rDNA clusters and/or the variable length of this satellite array inside IGS regions. Surprisingly, in *A. urticae*, all the rDNA unit elements showed even coverage including the AuSat region (fig. 4*c* and supplementary Tab. S5, Supplementary Material online). This discrepancy between results obtained through Illumina and PacBio reads may represent another interpopulational variability in this species in the repeat content (see above).

## Discussion

The arrays of major rRNA genes have become a very popular cytogenetic marker in comparative studies of karyotype evolution. Distribution of major rDNA can be relatively stable or rather dynamic in various taxa (Nguyen et al. 2010; Cabral-de-Mello et al. 2011; García-Souto et al. 2016; Perumal et al. 2017), and even intraspecific variability was observed (Baumgärtner et al. 2014; Šíchová et al. 2015; Ferretti et al. 2019). Changes in number and localization of rDNA loci have been ascribed to sequence homogeneity maintained by gene conversion (reviewed in Eickbush and Eickbush 2007) and chromosomal rearrangements mediated by ectopic recombination (Nguyen et al. 2010; Cabral-de-Mello et al. 2011; Ferretti et al. 2019), transposition (Raskina et al. 2004, 2008), or integration of extrachromosomal circular rDNA (ecc-rDNA; Proux-Wéra et al. 2013). However, none of the proposed mechanisms of rDNA mobility have been studied in detail so far.

To determine the position and number of rDNA clusters, only one probe, often 18S rDNA, is hybridized on chromosomes as a representative of the whole major rDNA unit. It is usually assumed that major rDNA units spread as a whole (Bueno et al. 2013). However, Ferretti et al. (2019) discovered high intraspecific and interspecific variability and independent mobility of each component of the major rDNA unit (18S, ITS1, 5.8S, ITS2, and 28S) in the genome of six different populations of a grasshopper *Abracris flavolineata*. A similar pattern was also observed in *Coregonus* fishes where both complete and partial rDNA units were detected by FISH mapping of individual rRNA genes (Symonová et al. 2013). Therefore, we physically mapped partial sequences of 18S and 28S rDNA to test whether rDNA spreads as a whole unit in the studied species. In *H. humuli* and *C. ohridella*, both 18S and 28S rDNA signals colocalized and covered significant portion of one chromosome pair (fig. 1*a* and *b*) as reported earlier (Provazníková et al. 2021). Seven and 11 rDNA loci were highlighted by both probes in *A. urticae* and *I. io*, respectively (fig. 1*c* and *d*), which is in agreement with known distribution of their major rDNA (Nguyen et al. 2010; Provazníková et al. 2021). Thus, it is reasonable to conclude that a complete major rDNA unit was amplified or spread into new loci in the species under study. This is further corroborated by coverage analyses carried out in *H. humuli*, *A. urticae*, and *I. io*, which showed similar read depth across their rDNA units (fig. 4*a*-*c* and supplementary table S5, Supplementary Material online).

For its dynamic evolution, rDNA has been often compared with repetitive sequences as arrays of rDNA are often found within heterochromatin (Lohe and Roberts 2000). Fragments of rDNA can be amplified into satellite-like tandem arrays (Lohe and Roberts 2000) and were found to be associated with satellites and other repeats (Jakubczak et al. 1991; Raskina et al. 2008; Symonová et al. 2013; Barbosa et al. 2015; Sember et al. 2018) which could mediate their spread (cf. Raskina et al. 2008; Nguyen et al. 2010; Proux-Wéra et al. 2013). Therefore, we clustered paired-end Illumina reads of species under study using the RE pipeline (Novák et al. 2010) and searched for association of identified repeats with major rRNA genes. The paired-end reads did not link major rDNA with any other clustered repeats in *C. ohridella* and *A. urticae*, although we could not have excluded that such repeats are present in either low frequencies or a distance bigger than the library insert size (see below). Yet, the association was recovered between major rDNA and Ty3/gypsy retrotransposon in *H. humuli* (Hh Ty3/gypsyA) and R2 element (IiR2) and a satellite (IiSat) in *I. io* (supplementary Table S1, Supplementary Material online).

Third-generation sequencing technologies have recently provided unprecedented insight into organization of repetitive sequences including rDNA genes (e.g., Sproul et al. 2020; Belser et al. 2021; Sims et al. 2021; Vondrak et al. 2021). We took advantage of *A. urticae* and *I. io* PacBio data recently released by the Darwin Tree of Life project and analyzed long reads, which contained major rDNA. The *I. io* data confirmed our previous results as the *I. io* long reads contained both the IiR2 and IiSat sequences (supplementary fig. S5, Supplementary Material online, fig. 3). Both proportion of long reads (supplementary fig. S5, Supplementary Material online) and coverage analysis (fig. 4*c* and supplementary table S5, Supplementary Material online) suggest that IiR2 is present in about one-third of rDNA units, which is in agreement with findings from other species (cf. Jakubczak et al. 1991; Zhou et al. 2013). In accordance with the results of RE, we found no MEs associated with rDNA in vast majority of reads in *A. urticae*. Small fraction of reads, which contained retrotransposon sequences of Ty3/Gypsy, R1 and R2 (supplementary fig. S6, Supplementary Material online), most likely corresponds to pseudogenes resulting from the birth-and-death process (cf. Martí et al. 2021). Yet, in contrast to the RE analysis, we found also satellite arrays (AuSat) in the IGS regions in majority of the *A. urticae* long reads bearing major rDNA (fig. 3 and supplementary fig. S6, Supplementary Material online). Surprisingly, different satellites are associated with major rDNA in the two closely related nymphalids. In both species, spacers notably varied in their length and the transcribed regions of the rDNA unit had in general a lower percentage of identical sites compared with *P. xylostella* with a single rDNA cluster (supplementary Table S4, Supplementary Material online) which points to a possible lack of concerted evolution. We hypothesize that this is due to a high number of major rDNA loci, which are not all transcriptionally active. Thus, they do not associate in nucleolus and evolve independently. Alternatively, the observed variation in spacer length could be ascribed to rDNA subtypes with tissue-specific expression or to mutations impairing chromatin modification enzymes (cf. Havlová et al. 2016).

In *H. humuli*, the Hh Ty3/gypsyA retrotransposon was inserted at the very end of the rDNA unit, at the junction between 28S rDNA gene and external transcribed spacer (supplementary fig. S1, Supplementary Material online, fig. 3). Mapping of its partial sequence by TSA FISH revealed its clear colocalization with rDNA (fig. 2*a*-*d*). Although we did not detect any other Hh Ty3/gypsyA loci, we cannot exclude that it is present elsewhere in the genome as interspersed repeats as the combined length of used probes is still under ca. 1300 bp detection limit of the TSA FISH protocol used (cf. Carabajal Paladino et al. 2014). Indeed, the coverage analysis suggests that abundance of MEs associated with rDNA is higher than the abundance of rRNA genes themselves (fig. 4*a* and supplementary table S5, Supplementary Material online). Furthermore, the Hh Ty3/gypsyA copy associated with major rDNA is nonautonomous. It represents only a portion of the corresponding RE cluster, which can be assembled into a complete retrotransposon with LTR repeats. Yet, the Hh Ty3/gypsyA sequences in IGS lack long terminal repeats and most of protein-coding domains (supplementary fig. S1, Supplementary Material online, fig. 3). Similar association between major rDNA and the Ty3/gypsy retrotransposon *Beon1* (Galadriel clade) was observed also in the beet *Beta vulgaris* where, however, the ME is inserted into 18S rRNA genes (Weber et al. 2013).

While short paired-end reads revealed only association between rDNA and Hh Ty3/gypsyA retrotransposon in *H. humuli*, long ONT reads showed that fragments of eight different elements were inserted in IGS (supplementary fig. S1, Supplementary Material online, fig. 3). Like the Hh Ty3/gypsyA (see above), none of the major rDNA–associated repeats is autonomous and thus cannot multiply on their own. However, their transmission is ensured by hitchhiking along with the indispensable rRNA gene family. It is not clear how expansion of IGS affected transcription of major rRNA genes. The IGS expanded to ca. 39 kb, and it is roughly on par with 45 kb long IGS of mouse (Grozdanov et al. 2003) and thus not necessarily detrimental. If the IGS expansion decreases expression of major rRNA genes, increase of the copy number would be favored, which would explain the extraordinary size of the *H. humuli* major rDNA cluster. Moreover, it is possible that rDNA did not actually spread along the chromosome. Rather, the total size of the array could have increased as major rDNA unit expanded due to insertion of repeats into IGS. Comparison of rDNA sequences between *H. humuli* and another hepialid *P. californicus* showed that the expansion of IGS is not shared across the family Hepialidae. *P. californicus* IGS and major rDNA transcription unit have in total only ca. 10 kb. Surprisingly, the IGS contains an array of insect telomeric motif TTAGG_(n)_ and TTATG microsatellite (supplementary fig. S2, Supplementary Material online, fig. 3; cf. Ruiz-Herrera et al. 2008; Scali et al. 2016; Sember et al. 2018). IGSs are known to contain repetitive motives, which usually do not correspond to MEs (Grozdanov et al. 2003; Havlová et al. 2016). However, association between rDNA and MEs has been reported with 5S being involved more often than 45S rDNA (da Silva et al. 2016; Yano et al. 2020). Insertions of MEs into IGS observed in *H. humuli* thus represent an interesting case of complex repeat organization (cf. Vondrak et al. 2021).

To pinpoint a mechanism responsible for changes in distribution of major rDNA in Lepidoptera, we took advantage of available long read sequencing data and compared the structure of major rDNA units between species with the highly derived rDNA distribution (see above) with three species with a single rDNA locus, namely, *P. xylostella*, *L. dispar*, and *S*. *frugiperda* (Nguyen et al. 2010; Provazníková et al. 2021). While we found MEs and/or satellite arrays at least in part of the IGS in all species with extraordinary major rDNA patterns but *C. ohridella* for which long reads have not been available, no repetitive sequences were associated with major rDNA in *S. frugiperda* (supplementary fig. S9, Supplementary Material online, fig. 3). However, satellite arrays (PxSat) of variable size were found in the IGS region in *P. xylostella* (supplementary fig. S7, Supplementary Material online, fig. 3) and analysis of *L. dispar* long reads revealed two types of R1 retrotransposon (supplementary fig. S8, Supplementary Material online) with 52.3% nucleotide identity in a small fraction of reads.

The R1 and R2 non-LTR retrotransposons are, along with the Pokey DNA transposon, among the few known rDNA-specific elements with insertion sites in the gene for 28S rRNA (Eickbush and Eickbush 2007; Elliott et al. 2013). The R elements represent one of the oldest groups of metazoan MEs (Kojima and Fujiwara 2005). They were described for the first time in a fruit fly, *Drosophila melanogaster*, by Roiha et al., (1981) and afterwards reported in many other animal phyla (reviewed in Eickbush and Eickbush 2015). Within the order Lepidoptera, the R1 and/or R2 elements have so far been detected only in several species, namely, *Bombyx mori* and *Bombyx mandarina* (both Bombycidae), *Manduca sexta* (Sphingidae), four representatives of the family Saturniidae, and *L. dispar* (Erebidae) (Fujiwara et al. 1984; Jakubczak et al. 1991; Kierzek et al. 2009). The latter is of particular interest as our observation of the two R1 retrotransposons and no R2 element in the same species suggests interpopulation differences and rapid turnover of the R1 and R2 retrotransposons. This is further supported by the absence of the R2 element in the Czech *A. urticae* compared with the British population. The R elements were found also in the nymphalids *A. urticae* and *I. io* with seven and eleven rDNA loci, respectively. It was proposed that the R2 retrotransposon plays an important role in maintaining rDNA copy numbers in *Drosophila* (Nelson et al. 2021). Yet, their absence in *S. frugiperda* and *P. xylostella* does not destabilize their rDNA loci and it seems unlikely that the R1 and R2 retrotransposons could mobilize rDNA in species under study. Although it was shown that the R1 and R2 retrotransposons can insert in the target site outside 28S rDNA in *B. mori* (Xiong et al. 1988), the IiR2 elements were not detected outside the major rDNA loci in *I. io* (fig. 2*b*, 4*b*). Moreover, insertion of the R1 and R2 elements into the 28S rDNA causes pseudogenization of the corresponding rDNA units (Long and Dawid 1979). The only other MEs associated with major rDNA were observed in *H. humuli.* However, these were not autonomous and their sequence diverged from those found in the rest of the genome. Thus, it seems unlikely that MEs could mediate spread of rDNA observed in some Lepidoptera (Provazníková et al. 2021) via transposition.

On the contrary, satellite arrays such as those found in IGS of nymphalids with a high number of rDNA loci, *A. urticae* and *I. io* (fig. 3), could facilitate homology-mediated spread of rDNA via either ectopic recombination or integration of extrachromosomal rDNA circles (Nguyen et al. 2010; Proux-Wéra et al. 2013; Sproul et al. 2020; Muirhead and Presgraves 2021). Yet, satellite arrays were associated with rDNA also in *P. xylostella* (fig. 3), which has only one rDNA locus. There is a difference between satellite DNA associated with rDNA in *P. xylostella* and the two nymphalids. In *P. xylostella*, we did not find any long reads bearing the PxSat without rDNA or at least partial IGS sequence. In both nymphalids, however, we identified more than 10 long reads bearing only the satellite arrays, with mean IGS and filtered read lengths being similar in all species which points to a presence of the satellites outside rDNA arrays. Unfortunately, coverage analyses are uninformative for satellites as there is a considerable variation in the number of their monomers per rDNA unit (supplementary fig. S7, Supplementary Material online). Inspection of the *A. urticae* genome assembly suggests that the AuSat is localized only in rDNA clusters and arrays adjacent to rDNA (supplementary fig. S6, Supplementary Material online), which suggests that rDNA spread into the AuSat loci. Interestingly, some satellite sequences seem to originate from IGS repeats (Macas et al. 2003; Jo et al. 2009; Guarrido-Ramos 2017). We hypothesize that the spread of rDNA in Lepidoptera is conditioned either by insertion of satellite sequence into IGS or by formation and spread of IGS-derived satellites outside rDNA loci. We cannot distinguish with certainty whether the rDNA spread occurred via ectopic recombination or integration of ecc-rDNA. Preferential spread of rDNA into terminal regions of lepidopteran chromosomes (Nguyen et al. 2010; Provazníková et al. 2021) seemingly supports ectopic recombination as its efficiency depends on proximity of homologous sequences to telomeres (Goldman and Lichten 1996; Nguyen et al. 2010), whereas ecc-rDNA could be integrated anywhere in the genome as long as a homologous sequence is present. However, it was shown that at least some types of ecc-DNA integrate preferentially near telomeres (Ruiz and Wahl 1990).

Little is known about satellite DNA in Lepidoptera, which has been studied in detail only in a dozen of species (Lu et al. 1994; Mandrioli et al. 2003; Mahendran et al. 2006; Věchtová et al. 2016; M. Dalíková et al. 2017b; Cabral-de-Mello et al. 2021; Haq et al. 2022). Yet, it seems that abundance of satellite DNA in lepidopteran genomes is very low with scattered distribution and possible enrichment on sex chromosomes (Cabral-de-Mello et al. 2021). This does not reflect distribution of rDNA in Lepidoptera (Nguyen et al. 2010; Provazníková et al. 2021). Yet, we cannot exclude that those satellites associated with major rDNA are limited to chromosome ends similar to *P. californicus*, which contains telomeric repeats in its IGS (supplementary fig. S4, Supplementary Material online).

Compared with other regions of rDNA arrays, IGS are rarely studied, and we thus cannot tell whether the observed complex association between rDNA and repetitive sequences (fig. 4) represents a common phenomenon. Our results show that long read sequencing is a valuable tool to study association of repeats including major rDNA as it provided more detailed information about major rDNA–associated repeats than analysis of short reads limited by library insert size. Moreover, the long read analysis provided better genomic representation compared with the genome assembly based on these long reads as seen in the *A. urticae* example (supplementary figs. S6 and S11, Supplementary Material online). Available target enrichment of major rDNA and other repeats for long read sequencing (McKinlay et al. 2021) could provide further insight into formation of complex repeat structures involving rDNA.

## Material and Methods

### Material

Specimens of all studied species were collected from wild populations. Females of *H. humuli* were collected in Bochov, Czech Republic, and let lay eggs in plastic containers. Hatched larvae were transferred and extensively reared in outdoor pots with planted carrots (*Daucus carota*). Larvae of *C. ohridella* were collected in České Budějovice, Czech Republic, from leaves of the horse chestnut, *Aesculus hippocastanum*. Specimens of *C. ohridella* were processed immediately after collection. Larvae of two nymphalids, *Inachis io* and *Aglais urticae*, were collected near Vrábče, Czech Republic. They were kept on the common nettle (*Urtica dioica*) in ambient conditions. Larvae of *P. californicus* were collected from the yellow bush lupine, *Lupinus arboreus*, in the Bodega Marine Reserve (California, USA). Larvae were used for chromosomal preparations and extraction of genomic DNA (gDNA) shortly after collection.

### Chromosomal Preparation

Chromosomal preparations were prepared by a spreading technique as described in Mediouni et al. (2004) and Dalíková et al. (2017a) with 10 min hypotonization of tissue. Meiotic and mitotic preparations were obtained from gonads of late larval instars of all four species. Afterwards, prepared slides were dehydrated in an ethanol series (70%, 80%, and 100% ethanol, 30 s each) and stored at −20 and −80 °C until further use. Remaining tissues were frozen for subsequent gDNA extraction.

### Genomic DNA Extraction

For downstream applications such as PCR, gDNA was extracted from larvae using NucleoSpin DNA Insect (Macherey-Nagel, Düren, Germany) according to manufacturer's protocol. To obtain high-molecular weight gDNA for NGS sequencing, gDNA was extracted from larvae using the MagAttract HMW DNA Kit (Qiagen, Hilden, Germany) or Nanobind Tissue Big DNA kit (Circulomics Inc., Baltimore, MD, USA) according to the manufacturer's protocol. Concentration of extracted samples was measured by Qubit 3.0 Fluorometer (Invitrogen, Carlsbad, CA, USA) and visualized on agarose gel. A single male larva was used as input material for all species but *C. ohridella*, for which 5–10 individuals (larvae and pupae) of both sexes were pooled because of their small size. A single male adult was used for extraction for Nanopore sequencing.

### Repeat Explorer Analysis

For analysis of repetitive DNA content, whole gDNA was sequenced on the Illumina platform generating either 150 bp pair-end reads from library with mean insert size 450 bp (Novogene Co. Ltd., Beijing, China) or 250 bp PE reads with the mean insert size 700 bp in case of *C. ohridella* (Genomics Core Facility, EMBL Heidelberg, Germany). The raw reads were quality filtered (average quality *Q* > 18) and trimmed from the 3′ end to a uniform length of 120 bp (230 bp for *C. ohridella*) by Trimmomatic 3.2 (Bolger et al. 2014). A random sample of two million (one million for *C. ohridella*) trimmed PE reads was generated by the RE utility sampleFasta.sh and analyzed by RE pipeline (version cerit-v0.3.1–2706) implemented in the Galaxy environment (http://repeatexplorer.org/) with automatic annotation via blastn and blastx using the REXdb Metazoan v3 database (Neumann et al. 2019). The resulting html files were searched for clusters annotated as major rDNA and their connection to other clusters.

### Probes for FISH Experiment

All mapped sequences were amplified by PCR using specific primers (for details, see table 1), purified from agarose gel, and cloned into Promega pGem T-Easy Vector (Promega, Madison, WI, USA). Selected clones were isolated using the Nucleo Spin Plasmid kit (Macherey-Nagel) and verified by sequencing (SEQme, Dobříš, Czech Republic).

**Table 1:** Primers Used for PCR Amplification of FISH Probe Templates and Labeling

Gene	Species	Forward/reverse primer	T_a_	Reference
*18S rDNA*	*Cydia pomonella*	CGATACCGCGAATGGCTCAATA/ACAAAGGGCAGGGACGTAATCAAC	58 °C	Fuková et al. 2005
*28S rDNA*	*Cydia pomonella*	GCAGATCTTGGTGGTAGTAGCA/GATGTACCGCCCCAGTCAAA	58 °C	This study
*Hh Ty3/GypsyA 1*	*Hepialus humuli*	AAATAAACTCTTAAAAGATGGAGT/TAATCTCCACTTCTTTTTCCC	58 °C	This study
*CL67contig10*				
*Hh Ty3/GypsyA 2*	*Hepialus humuli*	TTCGATTGAGGGTGATAGGCG/TCTCAAGCCTATCCAATCGCA	58 °C	This study
*CL67contig3*				
*Hh Ty3/GypsyA 3*	*Hepialus humuli*	TCTTGATCCTGGGTCTTTTACGTT/CGCGCTATTGGTAGTGTGCT	58 °C	This study
*CL67contig3*				
*IioR2 CL27contig8*	*Inachis io*	CCCAACAGAGAACACCCTCTC/GTGTTGGGGGATAGCAGGAAA	58 °C	This study
Iio satellite	*Inachis io*	GTAAACTTCGATTCCACAATACACGA/CTGTTATATTTCAAATGCAATGATCGA	58 °C	This study

Fragments of 18S and 28S rRNA genes were amplified from gDNA of codling moth, *Cydia pomonella* (Tortricidae) (cf. Nguyen et al. 2010). To obtain probes, these fragments were reamplified by PCR from plasmids using specific primers (table 1), purified by Wizard® SV Gel and PCR Clean-Up System (Promega), and labeled using nick translation protocol by Kato et al. (2006) with modifications described in Dalíková et al. (2017a). The 20 *µ*l labeling reaction contained 1 *µ*g DNA; 0.5 mM each dATP, dCTP, and dGTP; 0.1 mM dTTP; 20 *µ*M labeled nucleotides; 1× nick translation buffer (50 mM Tris–HCl, pH 7.5; 5 mM MgCl2; 0.005% BSA); 10 mM β-mercaptoethanol; 2.5× 10^−4^ U DNase I; and 1 U DNA polymerase I (both Thermo Fisher Scientific, Waltham, MA). The reaction was incubated at 15 °C for 45 min and enzymes were inactivated at 70 °C for 10 min. The 18S rDNA probe was labeled either with biotin-16-dUTP (Roche Diagnostics, Basel, Switzerland) or DNP-11-dUTP (Jena Bioscience, Jena, Germany), and the 28S rDNA probe was labeled by digoxigenin-11-dUTP (Roche Diagnostics) or fluorescein-12-dUTP (Jena Bioscience).

Three sequence fragments of Hh Ty3/GypsyA (CL67 contig 3 and 10) found in *H. humuli* and one of the R2 element (CL27 contig 8) and satellite (CL37 contig 4) found in *I. io* were also cloned as described above and separately labeled by PCR using plasmid DNA as template according to Provazníková et al. (2021). The 25 *µ*l labeling reaction contained 1–10 ng template plasmid DNA; 1× Ex Taq buffer; 1 mM each of dATP, dCTP, and dGTP; 0.36 mM dTTP; 0.62 mM labeled nucleotides of fluorescein-12-ddUTP (Jena Biosciences); 5 *µ*mol of each specific primer (table 1); and 0.25 U TaKaRa Ex Taq DNA polymerase (TaKaRa, Otsu, Japan). The resulting labeled probes were purified using Sephadex gel filtration (Illustra Sephadex G-50 fine DNA grade).

### FISH with 18S and 28S rDNA Probes

Indirect FISH was carried out according to Fuková et al. (2005) and Zrzavá et al. (2018) with some modifications and using two probes, biotin-labeled 18S rDNA probe and digoxigenin-labeled 28S rDNA probe, simultaneously. This technique was used to localize 18S and 28S rDNA genes in genome of *H. humuli*, *C. ohridella*, and *A. urticae*. The slides were dehydrated in an ethanol series (70, 80, and 100% ethanol, 30 s each) and pretreated with RNase A (200 ng/*µ*l) in 2× SSC at 37 °C for 1 h, washed twice in 2× SSC at RT for 5 min each, and incubated in 5×Denhardt's solution at 37 °C for 30 min. After, slides were denatured in 70% formamide in 2× SSC at 68 °C for 3.5 min and immediately dehydrated in an ethanol series (cold 70% for 1 min and 80% and 100% for 30 s each). Hybridization probe mix containing 10% dextran sulfate, 50% deionized formamide, 25 *µ*g of sonicated salmon sperm, and 50 ng of each probe in 2× SSC in final volume of 10 *µ*l was denatured at 90 °C for 5 min and immediately placed on ice for 2 min. Afterwards, hybridization probe mix was applied on the slide, covered by cover slip, and placed into a humid chamber. Hybridization was carried out at 37 °C overnight (12–16 h).

Next day, slides were incubated three times in 50% formamide in 2× SSC and followed by three washes in 2× SSC, both at 46 °C for 5 min. Slides were then washed three times with 0.1× SSC at 62 °C for 5 min and once in 1% Triton X in 4× SSC at RT for 10 min. The slides were blocked with 2.5% BSA in 4× SSC at RT for 30 min, incubated with anti-DIG1 (mouse anti-digoxigenin, 1:100, Roche Diagnostics, Basel, Switzerland) and streptavidin Cy3 conjugate (1:1000, Jackson ImmunoRes. Labs. Inc., West Grove, PA, USA) in 2.5% BSA in 4× SSC at 37 °C for 1 h, and washed three times with 1% Triton X in 4× SSC at 37 °C for 3 min each. To amplify the signals, the last three steps were repeated twice, firstly with anti-DIG2 (sheep anti-mouse Ig digoxigenin conjugate, 1:200, Merck Millipore, Billerica, MA, USA) and anti-streptavidin (1:25, Vector Labs. Inc, Burlingame, CA, USA) and secondly with anti-DIG3 (sheep anti-digoxigenin fluorescein conjugate, 1:200, Roche Diagnostics) and streptavidin-Cy3 conjugate (1:1000, Jackson ImmunoRes. Labs. Inc.). After the last washing step, slides were incubated in 1% Kodak PhotoFlo at RT for 1 min and counterstained with 0.5 mg/ml DAPI (4′,6-diamidino-2-phenylindole, Sigma-Aldrich, St. Louis, MO, USA) in antifade based on DABCO (1,4-diazabicyclo[2.2.2]-octane; Sigma–Aldrich).

### Double TSA FISH

Double FISH with tyramide signal amplification (double TSA FISH) was performed according to Carabajal Paladino et al. (2014) with some modifications. Due to its high sensitivity, double TSA FISH was employed to localize 18S and 28S rDNA genes in the *I. io* genome, and ME sequences and 18S rDNA gene in genomes of *I. io* and *H. humuli*. Briefly, slides were dehydrated in an ethanol series (70%, 80%, and 100% ethanol, 30 s each) and pretreated with 50 *µ*g/ml pepsin in 0.01 M HCl at 37 °C for 10 min, 1% H_2_O_2_ in 1× PBS at RT for 30 min, and in RNase A (100 *µ*g/ml) in 1× PBS at 37 °C for 1 h. After each pretreatment, the slides were washed three times in 1× PBS at RT for 5 min each washing. After the last washing, slides were incubated in 5× Denhardt's solution at 37 °C for 30 min. Directly after the last incubation, 50 *µ*l of hybridization probe mix containing 10% dextran sulphate, 50% deionized formamide, and 10–20 ng of each probe in 2× SSC was applied onto the slide, covered by a cover slip, and incubated at 70 °C for 5 min. Afterwards, slides were placed into the humid chamber and hybridized at 37 °C overnight (12–16 h). In *I. io* experiments, the 18S rDNA probe (10–20 ng) labeled with dinitrophenyl (DNP) was used with fluorescein-labeled R2 probe (10–20 ng), or fluorescein-labeled 28S rDNA probe (10–20 ng). In case of *H. humuli*, a combination of three fluorescein-labeled ME probes (Hh Ty3/GypsyA 1–3, 5–10 ng each) and the 18S rDNA probe (10–20 ng) was used.

The next day, slides were incubated three times for 5 min in 50% formamide in 2× SSC at 46 °C each, washed three times in 2× SSC at 46 °C for 5 min each and in 0.1× SSC at 62 °C for 5 min each, and washed once in 1× TNT at RT for 5 min. The slides were blocked in TNB buffer at RT for 30 min and incubated with anti-fluorescein-HRP Conjugate (PerkinElmer) in TNB (diluted 1:1000) at RT for 1 h. Afterwards, the slides were washed three times in 1× TNT at RT for 5 min each and incubated with TSA Plus Fluorescein (PerkinElmer) according to the manual at RT for 3–15 min (3–5 min in *H. humuli* and *C. ohridella* and 10–15 min for *I.io*) and washed again three times in 1× TNT at RT for 5 min each. To perform the second round of detection and to quench peroxidase activity from previous steps, slides were incubated in 1% H_2_O_2_ in 1× PBS at RT for 30 min. Next, the slides were washed three times in 1× TNT at RT for 5 min each and the amplification steps were repeated using anti-DNP-HRP Conjugate (PerkinElmer) and TSA Plus Cyanine 3 (PerkinElmer). After the last washing step, the slides were incubated in 1% Kodak PhotoFlo at RT for 1 min and counterstained with 0.5 mg/ml DAPI (Sigma–Aldrich) in antifade DABCO (Sigma–Aldrich).

### Microscoping and Image Processing

Chromosome preparations from FISH experiments were observed by a Zeiss Axioplan 2 microscope (Carl Zeiss, Jena, Germany) equipped with appropriate fluorescence filter sets. An Olympus CCD monochrome camera XM10 equipped with cellSens 1.9 digital imaging software (Olympus Europa Holding, Hamburg, Germany) was used to record and capture black-and-white pictures. Images were captured separately for each fluorescent dye and then pseudocolored and superimposed with Adobe Photoshop CS4, version 11.0.

### Long Read Sequencing and Analysis

High-molecular weight DNA from *H. humuli* was enriched for fragments longer than 10 kb by Short Read Eliminator (Circulomics Inc.). The library was prepared by Ligation Sequencing Kit SQK-LSK110 (Oxford Nanopore Technologies, Oxford, UK) according to the manufacture's protocol and therein recommended third-party consumables. The library was snap frozen and stored over night at −70 °C and then sequenced using flowcell R10.3 and MinION Mk1B (Oxford Nanopore Technologies). Reads were base called by guppy 4.4.1. with a high-accuracy flip-flop algorithm. The data were filtered for reads 15 kb and longer with a quality score over 10 using NanoFilt (De Coster et al. 2018).

Quality and length filtered reads were searched for the presence of major rDNA using blastn. Reads containing at least 1,000 bp of the *H. humuli* major rDNA unit were assembled by Flye 2.8 (Kolmogorov et al. 2019) using minimal overlap of 8 kb. The annotation of MEs was done by RepeatMasker 4.1.2-p1 (Smit et al. 2013) protein-based masking. Tandem repeats were identified based on self Dotplot implemented in Geneious 11.1.5. Consensus sequences of all identified ME fragments together with a major rDNA unit were mapped to individual rDNA bearing nanopore reads using minimap2 (Li, 2018) with appropriate preset. The presence and relative localization of individual elements was evaluated via R script (R version 4.2.1 in Rstudio version 1.4.1103) deposited in the Dryad Digital Repository (Dalíková et al. 2022). Only regions with a mapping quality of at least 20 were considered.

*P. californicus* gDNA was sequenced on Oxford Nanopore platform in Novogene Co. Ltd. PacBio HiFi reads of *I. io* (project PRJEB42130), *A. urticae* (project PRJEB42112), and *P. xylostella* (project PRJEB48395) were obtain through the Darwin Tree of Life project (http://www.darwintreeoflife.org). PacBio CLR data were obtained from the Sequence Read Archive (SRA) database (*S. frugiperda* SRR12642577; *L. dispar* SRR13505170-6, SRR13505182-3, and SRR13505187). Further, the reads were processed same as in *H. humuli* except for the HiFi reads, which were not quality filtered.

A similar approach to detect rDNA and associated repetitive DNA was used also in *A. urticae* chromosomal level genome assembly (Bishop et al. 2021) (ENA acc. No. PRJEB41896).

For the analysis of intragenomic sequence variability of rDNA genes, we extracted all sequences bearing rDNA from HiFi PacBio reads which did not contain R1 or R2 element and we used the clustering method implemented in program CD-HIT (version 4.6.1) (Fu et al. 2012). Furthermore, we aligned the rDNA sequences to the consensus using minimap2 (Li, 2018) and count the number of identical sites and average percentages of identity from the alignment in Geneious 11.1.5.

Coverage analysis was done by aligning genomic Illumina sequencing reads from *H. humuli*, *I. io*, and *A. urticae* to consensus sequences, using Bowtie2 aligner (Langmead and Salzberg 2012; Langmead et al. 2019). The consensus sequences used for this analysis were generated in Geneious 11.1.5 by overlapping the contigs of clusters and superclusters containing rDNA identified by RE analysis and/or assembled from long reads by Flye 2.8 assembler. Coverage values were obtained using samtools depth (v 1.10) (Li et al., 2009) and plotted using a script in R (R version 4.1.0 in Rstudio Workbench Version 1.4.1717-3). Mean coverage of defined annotation blocks as seen in figure 4 was computed using R and is in supplementary tables S5, Supplementary Material online.

## Supplementary Material

Supplementary data are available at *Genome Biology and Evolution* online (http://www.gbe.oxfordjournals.org/).

## Supplementary Material

evad090_Supplementary_Data

## Data Availability

Sequencing data generated in this study were deposited in the NCBI Sequence read archive under Bioproject reg. no. PRJNA737195. Long reads bearing rDNA of species under study, assemblies of their rDNA units, and R codes used for analyses were deposited in the Dryad Digital Repository under doi: 10.5061/dryad.gmsbcc2qj.

## References

[evad090-B1] Ahola V, et al 2014. The Glanville fritillary genome retains an ancient karyotype and reveals selective chromosomal fusions in Lepidoptera. Nat Commun. 5:4737. 25189940 10.1038/ncomms5737 PMC4164777

[evad090-B2] Barbosa P, et al 2015. Identification and chromosome mapping of repetitive elements in the *Astyanax scabripinnis* (Teleostei: Characidae) species complex. *Genetica*. 143:55–62. 25549800 10.1007/s10709-014-9813-2

[evad090-B3] Baumgärtner L, Paiz LM, Zawadzki CH, Margarido VP, Portela Castro ALB. 2014. Heterochromatin polymorphism and physical mapping of 5S and 18S ribosomal DNA in four populations of *Hypostomus strigaticeps* (Regan, 1907) from the Paraná River basin, Brazil: evolutionary and environmental correlation. *Zebrafish*. 11:479–487. 25237984 10.1089/zeb.2014.1028

[evad090-B4] Bedo DG . 1984. Karyotypic and chromosome banding studies of the potato tuber moth, *Phthorimaea operculella* (Zeller) (Lepidoptera, Gelechiidae). Can J Genet Cytol. 26:141–145.

[evad090-B5] Belser C, et al 2021. Telomere-to-telomere gapless chromosomes of banana using nanopore sequencing. Commun Biol. 4:1047. 34493830 10.1038/s42003-021-02559-3 PMC8423783

[evad090-B6] Bishop G, et al 2021. The genome sequence of the small tortoiseshell butterfly, *Aglais urticae* (linnaeus, 1758). Wellcome Open Res. 6:233. 10.12688/wellcomeopenres.17204.1 PMC9372638 36072556

[evad090-B7] Bolger AM, Lohse M, Usadel B. 2014. Trimmomatic: a flexible trimmer for Illumina sequence data. Bioinformatics. 30:2114–2120. 24695404 10.1093/bioinformatics/btu170 PMC4103590

[evad090-B8] Bueno D, Palacios-Gimenez OM, Cabral-de-Mello DC. 2013. Chromosomal mapping of repetitive DNAs in the grasshopper *Abracris flavolineata* reveal possible ancestry of the B chromosome and H3 histone spreading. PLoS ONE. 8:e66532. 23826099 10.1371/journal.pone.0066532 PMC3694960

[evad090-B9] Cabral-de-Mello DC, Oliveira SG, de Moura RC, Martins C. 2011. Chromosomal organization of the 18S and 5S rRNAs and histone H3 genes in Scarabaeinae coleopterans: insights into the evolutionary dynamics of multigene families and heterochromatin. BMC Genet. 12:88. 21999519 10.1186/1471-2156-12-88 PMC3209441

[evad090-B10] Cabral-de-Mello DC, Zrzavá M, Kubíčková S, Rendón P, Marec F. 2021. The role of satellite DNAs in genome architecture and sex chromosome evolution in Crambidae moths. Front Genet. 12:661417. 33859676 10.3389/fgene.2021.661417 PMC8042265

[evad090-B11] Carabajal Paladino LZ, et al 2019. Sex chromosome turnover in moths of the diverse superfamily Gelechioidea. Genome Biol Evol. 11:1307–1319. 31028711 10.1093/gbe/evz075 PMC6486803

[evad090-B12] Carabajal Paladino LZ, Nguyen P, Šíchová J, Marec F. 2014. Mapping of single-copy genes by TSA-FISH in the codling moth, *Cydia pomonella*. BMC Genet. 15:S15. 25471491 10.1186/1471-2156-15-S2-S15 PMC4255786

[evad090-B13] Cheng T, et al 2017. Genomic adaptation to polyphagy and insecticides in a major East Asian noctuid pest. Nat Ecol Evol. 1:1747–1756. 28963452 10.1038/s41559-017-0314-4

[evad090-B14] Dalíková M, et al 2017a. New insights into the evolution of the W chromosome in Lepidoptera. J Hered. 108:709–719. 28992287 10.1093/jhered/esx063

[evad090-B15] Dalíková M, Provazníková I, Provazník J, Grof-Tisza P, Pepi A, Nguyen P. 2022. The role of repetitive DNA in re-patterning of major rDNA clusters in Lepidoptera, Dryad, Dataset. 10.5061/dryad.gmsbcc2qj PMC10257491 37226278

[evad090-B16] Dalíková M, Zrzavá M, Kubíčková S, Marec F. 2017b. W-enriched satellite sequence in the Indian meal moth, *Plodia interpunctella* (Lepidoptera, Pyralidae). Chromosome Res. 25:241–252. 28500471 10.1007/s10577-017-9558-8

[evad090-B17] da Silva M, Barbosa P, Artoni RF, Feldberg E. 2016. Evolutionary dynamics of 5S rDNA and recurrent association of transposable elements in electric fish of the family Gymnotidae (Gymnotiformes): the case of *Gymnotus mamiraua*. Cytogenet Genome Res. 149:297–303. 27750255 10.1159/000449431

[evad090-B18] De Coster W, D’Hert S, Schultz DT, Cruts M, Van Broeckhoven C. 2018. Nanopack: visualizing and processing long-read sequencing data. Bioinformatics. 34:2666–2669. 29547981 10.1093/bioinformatics/bty149 PMC6061794

[evad090-B19] de Sene VF, et al 2015. Mapping of the retrotransposable elements *Rex1* and *Rex3* in chromosomes of *Eigenmannia* (Teleostei, Gymnotiformes, Sternopygidae). Cytogenet Genome Res. 146:319–324. 26559509 10.1159/000441465

[evad090-B20] Eickbush TH, Eickbush DG. 2007. Finely orchestrated movements: evolution of the ribosomal RNA genes. Genetics. 175:477–485. 17322354 10.1534/genetics.107.071399 PMC1800602

[evad090-B21] Eickbush TH, Eickbush DG. 2015. Integration, regulation, and long-term stability of R2 retrotransposons. Microbiol Spectr. 3:2. 10.1128/microbiolspec.MDNA3-0011-2014 PMC4498411 26104703

[evad090-B22] Elliott TA, Stage DE, Crease TJ, Eickbush TH. 2013. In and out of the rRNA genes: characterization of *Pokey* elements in the sequenced *Daphnia* genome. Mob DNA. 4:20. 24059783 10.1186/1759-8753-4-20 PMC3849761

[evad090-B23] Ferretti ABSM, et al 2019. How dynamic could be the 45S rDNA cistron? An intriguing variability in a grasshopper species revealed by integration of chromosomal and genomic data. Chromosoma. 128:165–175. 31111199 10.1007/s00412-019-00706-8

[evad090-B24] Fiore-Donno AM, et al 2012. 18S rDNA phylogeny of *Lamproderma* and allied genera (Stemonitales, Myxomycetes, Amoebozoa). PLoS ONE. 7:e35359. 22530009 10.1371/journal.pone.0035359 PMC3329430

[evad090-B25] Fu L, Niu B, Zhu Z, Wu S, Li W. 2012. CD-HIT: accelerated for clustering the next-generation sequencing data. Bioinformatics. 28:3150–3152. 23060610 10.1093/bioinformatics/bts565 PMC3516142

[evad090-B26] Fujiwara H, et al 1984. Introns and their flanking sequences of *Bombyx mori* rDNA. Nucl Acids Res. 12:6861–6869. 6091041 10.1093/nar/12.17.6861 PMC320122

[evad090-B27] Fuková I, Nguyen P, Marec F. 2005. Codling moth cytogenetics: karyotype, chromosomal location of rDNA, and molecular differentiation of sex chromosomes. Genome. 48:1083–1092. 16391677 10.1139/g05-063

[evad090-B28] García-Souto D, Pérez-García C, Kendall J, Pasantes J. 2016. Molecular cytogenetics in trough shells (Mactridae, Bivalvia): divergent GC-rich heterochromatin content. Genes (Basel). 7:47. 27537915 10.3390/genes7080047 PMC4999835

[evad090-B29] García S, Kovařík A, Leitch AR, Garnatje T. 2017. Cytogenetic features of rRNA genes across land plants: analysis of the Plant rDNA database. Plant J. 89:1020–1030. 27943584 10.1111/tpj.13442

[evad090-B30a] Garrido-Ramos MA . 2017. Satellite DNA: An Evolving Topic. Genes (Basel). 8:230. 28926993 10.3390/genes8090230 PMC5615363

[evad090-B30] Goldman ASH, Lichten M. 1996. The efficiency of meiotic recombination between dispersed sequences in *Saccharomyces cerevisiae* depends upon their chromosomal location. Genetics. 144:43–55. 8878672 10.1093/genetics/144.1.43 PMC1207516

[evad090-B31] Grozdanov P, Georgiev O, Karagyozov L. 2003. Complete sequence of the 45-kb mouse ribosomal DNA repeat: analysis of the intergenic spacer. Genomics. 82:637–643. 14611805 10.1016/s0888-7543(03)00199-x

[evad090-B32] Haq IU, et al 2022. Satellitome analysis and transposable elements comparison in geographically distant populations of *Spodoptera frugiperda*. Life (Basel). 12:521. 35455012 10.3390/life12040521 PMC9026859

[evad090-B33] Havlová K, et al 2016. Variation of 45S rDNA intergenic spacers in *Arabidopsis thaliana*. Plant Mol Biol. 92:457–471. 27531496 10.1007/s11103-016-0524-1

[evad090-B34] Ingle J, Timmis JN, Sinclair J. 1975. The relationship between satellite deoxyribonucleic acid, ribosomal ribonucleic acid gene redundancy, and genome size in plants. Plant Physiol. 55:496–501. 16659109 10.1104/pp.55.3.496 PMC541645

[evad090-B35] Jakubczak JL, Burke WD, Eickbush TH. 1991. Retrotransposable elements RI and R2 interrupt the rRNA genes of most insects. Proc Natl Acad Sci U S A. 88:3295–3299. 1849649 10.1073/pnas.88.8.3295 PMC51433

[evad090-B36] Jo SH, et al 2009. Evolution of ribosomal DNA-derived satellite repeat in tomato genome. BMC Plant Biol. 9:42. 19351415 10.1186/1471-2229-9-42 PMC2679016

[evad090-B37] Kato A, et al 2006. Sensitive fluorescence *in situ* hybridization signal detection in maize using directly labeled probes produced by high concentration DNA polymerase nick translation. Biotechnic Histochem. 81:71–78. 10.1080/10520290600643677 16908431

[evad090-B38] Kierzek E, et al 2009. Secondary structures for 5′ regions of R2 retrotransposon RNAs reveal a novel conserved pseudoknot and regions that evolve under different constraints. J Mol Biol. 390:428–442. 19397915 10.1016/j.jmb.2009.04.048 PMC2728621

[evad090-B39] Kobayashi T . 2011. Regulation of ribosomal RNA gene copy number and its role in modulating genome integrity and evolutionary adaptability in yeast. Cell Mol Life Sci. 68:1395–1403. 21207101 10.1007/s00018-010-0613-2 PMC3064901

[evad090-B40] Kojima KK, Fujiwara H. 2005. Long-term inheritance of the 28S rDNA-specific retrotransposon R2. Mol Biol Evol. 22:2157–2165. 16014872 10.1093/molbev/msi210

[evad090-B41] Kolmogorov M, Yuan J, Lin Y. 2019. Assembly of long, error-prone reads using repeat graphs. Nat Biotechnol. 37:540–546. 30936562 10.1038/s41587-019-0072-8

[evad090-B42] Langmead B, Salzberg SL. 2012. Fast gapped-read alignment with Bowtie 2. Nat Methods. 9:357–359. 22388286 10.1038/nmeth.1923 PMC3322381

[evad090-B43] Langmead B, Wilks C, Antonescu V, Charles R. 2019. Scaling read aligners to hundreds of threads on general-purpose processors. Bioinformatics. 35:421–432. 30020410 10.1093/bioinformatics/bty648 PMC6361242

[evad090-B44] Li H, et al 2009. The sequence Alignment/Map format and SAMtools. Bioinformatics. 25:2078–2079. 19505943 10.1093/bioinformatics/btp352 PMC2723002

[evad090-B45] Li H . 2018. Minimap2: pairwise alignment for nucleotide sequences. Bioinformatics. 34:3094–3100. 29750242 10.1093/bioinformatics/bty191 PMC6137996

[evad090-B46] Lohe AR, Roberts PA. 2000. Evolution of DNA in heterochromatin: the *Drosophila melanogaster* sibling species subgroup as a resource. Genetica. 109:125–130. 11293787 10.1023/a:1026588217432

[evad090-B47] Long EO, Dawid IB. 1979. Expression of ribosomal DNA insertions in *Drosophila melanogaster*. Cell. 18:1185–1196. 117903 10.1016/0092-8674(79)90231-9

[evad090-B48] Long EO, Dawid IB. 1980. Repeated genes in eukaryotes. Annu Rev Biochem. 49:727–764. 6996571 10.1146/annurev.bi.49.070180.003455

[evad090-B49] Lu Y, Kochert GD, Isenhour DJ, Adang MJ. 1994. Molecular characterization of a strain-specific repeated DNA sequence in the fall armyworm *Spodoptera frugiperda* (Lepidoptera: Noctuidae). Insect Mol Biol. 3:123–130. 7987522 10.1111/j.1365-2583.1994.tb00159.x

[evad090-B50] Lukhtanov V . 2015. The blue butterfly *Polyommatus (Plebicula) atlanticus* (Lepidoptera, Lycaenidae) holds the record of the highest number of chromosomes in the non-polyploid eukaryotic organisms. Comparat Cytogenet. 9:683–690. 10.3897/CompCytogen.v9i4.5760 PMC4698580 26753083

[evad090-B51] Macas J, Navrátilová A, Mészáros T. 2003. Sequence subfamilies of satellite repeats related to rDNA intergenic spacer are differentially amplified on Vicia sativa chromosomes. Chromosoma. 112:152–158. 14579131 10.1007/s00412-003-0255-3

[evad090-B52] Mahendran B, Acharya C, Dash R, Ghosh SK, Kundu SC. 2006. Repetitive DNA in tropical tasar silkworm *Antheraea mylitta*. Gene. 370:51–57. 16455212 10.1016/j.gene.2005.11.010

[evad090-B53] Mandrioli M, Manicardi GC, Marec F. 2003. Cytogenetic and molecular characterization of the MBSAT1 satellite DNA in holokinetic chromosomes of the cabbage moth, *Mamestra brassicae* (Lepidoptera). Chromosome Res. 11:51–56. 12675305 10.1023/a:1022058032217

[evad090-B54] Martí E, et al 2021. Cytogenomic analysis unveils mixed molecular evolution and recurrent chromosomal rearrangements shaping the multigene families on *Schistocerca* grasshopper genomes. Evolution. 75:2027–2041. 34155627 10.1111/evo.14287

[evad090-B55] McKinlay A, Fultz D, Wang F, Pikaard CS. 2021. Targeted enrichment of rRNA gene tandem arrays for ultra-long sequencing by selective restriction endonuclease digestion. Front Plant Sci. 12:656049. 33995452 10.3389/fpls.2021.656049 PMC8113872

[evad090-B56] Mediouni J, Fuková I, Frydrychová R, Dhouibi MH, Marec F. 2004. Karyotype, sex chromatin and sex chromosome differentiation in the carob moth, *Ectomyelois ceratoniae* (Lepidoptera: Pyralidae). Caryologia. 57:184–194.

[evad090-B57] Muirhead CA, Presgraves DC. 2021. Satellite DNA-mediated diversification of a sex-ratio meiotic drive gene family in *Drosophila*. Nat Ecol Evol. 5:1604–1612. 34489561 10.1038/s41559-021-01543-8 PMC11188575

[evad090-B58] Nelson JO, Slicko A, Yamashita YM. 2023. The retrotransposon R2 maintains *Drosophila* ribosomal DNA repeats. Proc Natl Acad Sci U S A. 120:e2221613120. 10.1073/pnas.2221613120 PMC10266012 37252996

[evad090-B59] Neumann P, Novák P, Hoštáková N, Macas J. 2019. Systematic survey of plant LTR-retrotransposons elucidates phylogenetic relationships of their polyprotein domains and provides a reference for element classification. Mob DNA. 10:1. 30622655 10.1186/s13100-018-0144-1 PMC6317226

[evad090-B60] Nguyen P, Sahara K, Yoshido A, Marec F. 2010. Evolutionary dynamics of rDNA clusters on chromosomes of moths and butterflies (lepidoptera). Genetica. 138:343–354. 19921441 10.1007/s10709-009-9424-5

[evad090-B61] Noller HF, Lancaster L, Mohan S, Zhou J. 2017. Ribosome structural dynamics in translocation: yet another functional role for ribosomal RNA. Quart Rev Biophys. 50:e12. 10.1017/S0033583517000117 29233224

[evad090-B62] Novák P, Neumann P, Macas J. 2010. Graph-based clustering and characterization of repetitive sequences in next-generation sequencing data. BMC Bioinformatics. 11:378. 20633259 10.1186/1471-2105-11-378 PMC2912890

[evad090-B63] Novák P, Neumann P, Pech J, Steinhaisl J, Macas J. 2013. Repeatexplorer: a Galaxy-based web server for genome-wide characterization of eukaryotic repetitive elements from next-generation sequence reads. Bioinformatics. 29:792–793. 23376349 10.1093/bioinformatics/btt054

[evad090-B64] Palacios-Gimenez OM, Castillo ER, Martí DA, Cabral-de-Mello DC. 2013. Tracking the evolution of sex chromosome systems in Melanoplinae grasshoppers through chromosomal mapping of repetitive DNA sequences. BMC Evol Biol. 13:167. 23937327 10.1186/1471-2148-13-167 PMC3751140

[evad090-B65] Perumal S, et al 2017. Elucidating the major hidden genomic components of the A, C, and AC genomes and their influence on *Brassica* evolution. Sci Rep. 7:17986. 29269833 10.1038/s41598-017-18048-9 PMC5740159

[evad090-B66] Poletto AB, et al 2010. Chromosome differentiation patterns during cichlid fish evolution. BMC Genet. 11:50. 20550671 10.1186/1471-2156-11-50 PMC2896337

[evad090-B67] Prins JD, Saitoh K. 2003. Karyology and sex determination. In: Kükenthal W, editors. Band 4: Arthropoda, 2 Hälfte: Insecta, Lepidoptera, moths and butterflies, Teilband/part 36, vol 2: morphology, physiology, and development. Berlin, Boston: DE GRUYTER. p. 449–468.

[evad090-B68] Prokopowich CD, Gregory TR, Crease TJ. 2003. The correlation between rDNA copy number and genome size in eukaryotes. Genome. 46:48–50. 12669795 10.1139/g02-103

[evad090-B69] Proux-Wéra E, Byrne KP, Wolfe KH. 2013. Evolutionary mobility of the ribosomal DNA array in yeasts. Genome Biol Evol. 5:525–531. 23419706 10.1093/gbe/evt022 PMC3622299

[evad090-B70] Provazníková I, et al 2021. Large-scale comparative analysis of cytogenetic markers across Lepidoptera. Sci Rep. 11:12214. 34108567 10.1038/s41598-021-91665-7 PMC8190105

[evad090-B71] Raskina O, Barber JC, Nevo E, Belyayev A. 2008. Repetitive DNA and chromosomal rearrangements: speciation-related events in plant genomes. Cytogenet Genome Res. 120:351–357. 18504364 10.1159/000121084

[evad090-B72] Raskina O, Belyayev A, Nevo E. 2004. Activity of the *En/Spm*-like transposons in meiosis as a base for chromosome repatterning in a small, isolated, peripheral population of *Aegilops speltoides* Tausch. Chromosome Res. 12:153–161. 15053485 10.1023/b:chro.0000013168.61359.43

[evad090-B73] Rego A, Marec F. 2003. Telomeric and interstitial telomeric sequences in holokinetic chromosomes of Lepidoptera: telomeric DNA mediates association between postpachytene bivalents in achiasmatic meiosis of females. Chromosome Res. 11:681–694. 14606630 10.1023/a:1025937808382

[evad090-B74] Robinson R . 1971. Lepidoptera genetics. Oxford: Pergamon Press.

[evad090-B75] Roiha H, Miller J, Woods L. 1981. Arrangements and rearrangements of sequences flanking the two types of rDNA insertion in *D. melanogaster*. Nature. 290:749–754. 6783966 10.1038/290749a0

[evad090-B76] Ruiz-Herrera A, Nergadze SG, Santagostino M, Giulotto E. 2008. Telomeric repeats far from the ends: mechanisms of origin and role in evolution. Cytogenet Genome Res. 122:219–228. 19188690 10.1159/000167807

[evad090-B77] Ruiz JC, Wahl GM. 1990. Chromosomal destabilization during gene amplification. Mol Cell Biol. 10:3056–3066. 2188107 10.1128/mcb.10.6.3056-3066.1990 PMC360670

[evad090-B78] Scacchetti PC, et al 2012. Molecular characterization and physical mapping of two classes of 5S rDNA in the genomes of *Gymnotus sylvius* and *G. inaequilabiatus* (Gymnotiformes, Gymnotidae). Cytogenet Genome Res. 136:131–137. 22285951 10.1159/000335658

[evad090-B79] Scali V, et al 2016. Co-localization of ribosomal and telomeric sequences in *Leptynia* (Insecta: Phasmatodea). Ital J Zool. 83:285–290.

[evad090-B80] Sember A, et al 2018. Dynamics of tandemly repeated DNA sequences during evolution of diploid and tetraploid botiid loaches (Teleostei: Cobitoidea: Botiidae). PLoS ONE. 13:e0195054. 29590207 10.1371/journal.pone.0195054 PMC5874072

[evad090-B81] Šíchová J, et al 2015. Dynamic karyotype evolution and unique sex determination systems in Leptidea wood white butterflies. BMC Evol Biol. 15:89. 25981157 10.1186/s12862-015-0375-4 PMC4436027

[evad090-B82] Šíchová J, et al 2016. Fissions, fusions, and translocations shaped the karyotype and multiple sex chromosome constitution of the northeast-Asian wood white butterfly, *Leptidea amurensis*. Biol J Linn Soc. 118:457–471.

[evad090-B83] Šíchová J, Nguyen P, Dalíková M, Marec F. 2013. Chromosomal evolution in tortricid moths: conserved karyotypes with diverged features. PLoS ONE. 8:e64520. 23717623 10.1371/journal.pone.0064520 PMC3663796

[evad090-B84] Silva DMZ de A, et al 2014. Delimiting the origin of a B chromosome by FISH mapping, chromosome painting and DNA sequence analysis in *Astyanax paranae* (Teleostei, Characiformes). PLoS ONE. 9:e94896. 24736529 10.1371/journal.pone.0094896 PMC3988084

[evad090-B85] Sims J, Sestini G, Elgert C, von Haeseler A, Schlögelhofer P. 2021. Sequencing of the *Arabidopsis* NOR2 reveals its distinct organization and tissue-specific rRNA ribosomal variants. Nat Commun. 12:387. 33452254 10.1038/s41467-020-20728-6 PMC7810690

[evad090-B86] Smit AFA, Hubley R, Green P. 2013. RepeatMasker Open-4.0.

[evad090-B87] Sochorová J, Garcia S, Gálvez F, Symonová R, Kovařík A. 2018. Evolutionary trends in animal ribosomal DNA loci: introduction to a new online database. Chromosoma. 127:141–150. 29192338 10.1007/s00412-017-0651-8 PMC5818627

[evad090-B88] Sproul JS, Barton LM, Maddison DR. 2020. Repetitive DNA profiles reveal evidence of rapid genome evolution and reflect species boundaries in ground beetles. Syst Biol. 69:1137–1148. 32267949 10.1093/sysbio/syaa030

[evad090-B89] Symonová R, et al 2013. Genome differentiation in a species pair of coregonine fishes: an extremely rapid speciation driven by stress-activated retrotransposons mediating extensive ribosomal DNA multiplications. BMC Evol Biol. 13:42. 23410024 10.1186/1471-2148-13-42 PMC3585787

[evad090-B90] Symonová R . 2019. Integrative rDNAomics—importance of the oldest repetitive fraction of the eukaryote genome. Genes (Basel). 10:345. 31067804 10.3390/genes10050345 PMC6562748

[evad090-B91] Van Nieukerken EJ, et al 2011. Order Lepidoptera Linnaeus, 1758. In: Zhang, Z.-Q. (Ed.) animal biodiversity: an outline of higher-level classification and survey of taxonomic richness. Zootaxa. 3148:212. 10.11646/zootaxa.3703.1.1 26146682

[evad090-B92] Van’t Hof AE, et al 2013. Linkage map of the peppered moth, *Biston betularia* (Lepidoptera, Geometridae): a model of industrial melanism. Heredity (Edinb). 110:283–295. 23211790 10.1038/hdy.2012.84 PMC3668655

[evad090-B93] Věchtová P, et al 2016. CpSAT-1, a transcribed satellite sequence from the codling moth, *Cydia pomonella*. Genetica. 144:385–395. 27236660 10.1007/s10709-016-9907-0

[evad090-B94] Vershinina AO, Anokhin BA, Lukhtanov VA. 2015. Ribosomal DNA clusters and telomeric (TTAGG)n repeats in blue butterflies (Lepidoptera, Lycaenidae) with low and high chromosome numbers. Comp Cytogenet. 9:161–171. 26140159 10.3897/CompCytogen.v9i2.4715 PMC4488964

[evad090-B95] Vítková M, Fuková I, Kubíčková S, Marec F. 2007. Molecular divergence of the W chromosomes in pyralid moths (Lepidoptera). Chromosome Res. 15:917–930. 17985203 10.1007/s10577-007-1173-7

[evad090-B96] Vondrak T, et al 2021. Complex sequence organization of heterochromatin in the holocentric plant *Cuscuta europaea* elucidated by the computational analysis of nanopore reads. Comput Struct Biotechnol. 19:2179–2189. 10.1016/j.csbj.2021.04.011 PMC8091179 33995911

[evad090-B97a] Weber B, et al 2013. Highly diverse chromoviruses of Beta vulgaris are classified by chromodomains and chromosomal integration. Mobile DNA. 4:8. 10.1186/1759-8753-4-8 PMC3605345 23448600

[evad090-B97] Wolf KW, Novak K, Marec F. 1997. Kinetic organization of metaphase I bivalents in spermatogenesis of Lepidoptera and Trichoptera species with small chromosome numbers. Heredity (Edinb). 79:35–143.

[evad090-B98] Wu S, Xiong J, Yu Y. 2015. Taxonomic resolutions based on 18S rRNA genes: a case study of subclass Copepoda. PLoS ONE. 10:e0131498. 26107258 10.1371/journal.pone.0131498 PMC4479608

[evad090-B99] Xiong Y, Burke WD, Jakubczak JL, Eickbush TH. 1988. Ribosomal DNA insertion elements R1Bm and R2Bm can transpose in a sequence specific manner to locations outside the 28S genes. Nucleic Acids Res. 16:10561–10573. 2849750 10.1093/nar/16.22.10561 PMC338924

[evad090-B100] Yano CF, et al 2020. Evolutionary dynamics of multigene families in *Triportheus* (Characiformes, Triportheidae): a transposon mediated mechanism? Front Mar Sci. 7:6.

[evad090-B101] Yasukochi Y, et al 2011. Isolation of BAC clones containing conserved genes from libraries of three distantly related moths: a useful resource for comparative genomics of Lepidoptera. J Biomed Biotechnol. 2011:1–6. 10.1155/2011/165894 PMC2992816 21127704

[evad090-B102] Zhou J, Eickbush MT, Eickbush TH. 2013. A population genetic model for the maintenance of R2 retrotransposons in rRNA gene loci. PLoS Genet. 9:e1003179. 23326244 10.1371/journal.pgen.1003179 PMC3542110

[evad090-B103] Zrzavá M, et al 2018. Sex chromosomes of the iconic moth *Abraxas grossulariata* (Lepidoptera, Geometridae) and its congener *A. sylvata*. Genes (Basel). 9:279. 29857494 10.3390/genes9060279 PMC6027526

