# Supplementary material for: The Role of Repetitive Sequences in Repatterning of Major Ribosomal DNA Clusters in Lepidoptera
Source: Genome Biol Evol. 2023 May 24;15(6):evad090. doi: 10.1093/gbe/evad090 (PMC10257491; doi:10.1093/gbe/evad090)
Supplement: evad090_Supplementary_Data [file evad090_supplementary_data.zip › Supplementary_Material_Legends.docx]

**Supplementary figures**

**Figure S1:** Overview of the number and position of 18S rDNA marker in haploid genomes of studied species adapted from Provazníková et al. (2021; doi:10.1038/s41598-021-91665-7) and references therein. Phylogenetic relationships are based on Kawahara et al. (2019; doi:10.1073/pnas.1907847116) and Wiemers et al. (2020; doi:10.3897/zookeys.938.50878). Note that Tineoidea are considered paraphyletic (6a, b). * Species included in this study (in bold). n.d.—not determined. F/M—female and male diploid chromosome numbers, if different. The figure was created in Adobe Illustrator 2020, version 24.0 ([www.adobe.com](http://www.adobe.com)).

**Figure S2:** Graph layouts generated by the RepeatExplorer pipeline corresponding to clusters comprised in superclusters with detected rDNA genes. Nodes represent individual Illumina reads and edges their sequence overlap.  **a**) *Cameraria* *ohridella*, **b**) *Inachis* *io*, **c**) *Aglais* *urticae*, and **d**) *Hepialus humulli*. Arrows indicate regions of graphs corresponding to satellite sequences as evidenced by their read density. rDNA genes and protein coding domains of mobile elements annotated by RepeatExplorer are colour coded. INT – integrase, RT – reverse transcriptase, PROT – protease, RH – Rnase H.

**Figure S3:** Visualization of major rDNA genes and associated repeats on *H. humuli* Oxford Nanopore reads. Only matches longer than 200 bp and with mapping quality over 20 were considered. Gray line represents the read length, coloured boxes correspond to the rDNA genes or individual detected repeats.

**Figure S4:** Visualization of major rDNA genes and associated repeats on *P. californicus* Oxford Nanopore reads. Only matches longer than 200 bp and with mapping quality over 20 were considered. Gray line represents the read length, coloured boxes correspond to the rDNA genes or individual detected repeats.

**Figure S5:** Visualization of major rDNA genes and associated repeats on *I. io* HiFi PacBio reads. Only matches longer than 200 bp and with mapping quality over 20 were considered. Gray line represents the read length, coloured boxes correspond to the rDNA genes or individual detected repeats.

**Figure S6:** Visualization of major rDNA genes and associated repeats on *A. urticae* HiFi PacBio reads. Only matches longer than 200 bp and with mapping quality over 20 were considered. Gray line represents the read length, coloured boxes correspond to the rDNA genes or individual detected repeats.

**Figure S7:** Visualization of major rDNA genes and associated satellite on *P. xylostella* PacBio reads. Only matches longer than 200 bp and with mapping quality over 20 were considered. Gray line represents the read length, coloured boxes correspond to the rDNA genes or PxSat.

**Figure S8:** Visualization of major rDNA genes and associated repeats on *L. dispar* PacBio reads. Only matches longer than 200 bp and with mapping quality over 20 were considered. Gray line represents the read length, coloured boxes correspond to the rDNA genes or individual detected repeats.

**Figure S9:** Visualization of major rDNA genes *S. frugiperda* PacBio reads. Only matches longer than 200 bp and with mapping quality over 20 were considered, no associated repeats were detected. Gray line represents the read length, coloured boxes correspond to the rDNA genes.

**Figure S10:** Distribution of AuSat (in *A. urticae*), IiSat (in *I. io*), and PxSat (in *P. xylostella*) arrays lengths in PacBio reads containing at least 500bp of major rDNA genes. Bold line represents median value, box corresponds to interquartile range, and the outlier values are represented by individual dots.

**Figure S11:** Visualization of major rDNA genes and associated repeats from the current *A. urticae* genome assembly. Only matches longer than 200 bp and with mapping quality over 20 were considered. Grey line represents the chromosomal length scaffolds with detected rDNA clusters, coloured boxes correspond to the rDNA genes or individual detected repeats.

**Figure S12:** Visualization of clusters and percentage of rDNA units corresponding to individual clusters obtained by CD-HIT program analysis. The analysis was performed with two thresholds, 80% (**a**) and 95% (**b**) identity.

**Supplementary Tables**

**Table S1:** Summary of basic characteristics of selected clusters from Repeat Explorer analysis in *H. humuli*, *A. urticae*, *I. io*, and *C. ohridella*

**Table S2**: Annotation of consensus sequences of complete rDNA unit as obtained from long reads assemblies.

**Table S3:** Length characterisation of PacBio reads and satellite arrays in *A. urticae*, *I. io*, and *P. xylostella*

**Table S4:** Diversity of 18S, ITS1, 5.8S, ITS2, and 28S sequences in *A. urticae*, *I. io*, and *P. xylostella* represented by percentage of identical sites average percentage of identity between consensus and individual rDNA copy obtained from PacBio HiFi reads.

**Table S5:** Summary of coverage analysis of individual elements in rDNA units in *H. humuli*, *A. urticae*, and *I. io*
