# Supplementary material for: The Role of Repetitive Sequences in Repatterning of Major Ribosomal DNA Clusters in Lepidoptera
Source: Genome Biol Evol. 2023 May 24;15(6):evad090. doi: 10.1093/gbe/evad090 (PMC10257491; doi:10.1093/gbe/evad090)
Supplement: evad090_Supplementary_Data [file evad090_supplementary_data.zip › SupplementaryMaterial_R2.pdf]

Supplementary Material

for

The role of repetitive sequences in re-patterning of major rDNA clusters in Lepidoptera

Martina Dalíková [1,2,$], Irena Provazníková [1,2,3,$], Jan Provazník [1,3], Patrick Grof-Tisza[4], Adam Pepi[5], and Petr Nguyen [1,2 *]

Affiliation:

1 Faculty of Science, University of South Bohemia, České Budějovice, Czech Republic

2 Institute of Entomology, Biology Centre CAS, České Budějovice, Czech Republic

3 Current address: European Molecular Biology Laboratory, Heidelberg, Germany

4 Institute of Biology, Laboratory of Evolutionary Entomology, University of Neuchâtel, Neuchâtel, Switzerland

5 Department of Biology, Tufts University, Medford MA USA

$ These authors contributed equally to this work

*** Correspondence to:**

Petr Nguyen

University of South Bohemia
Faculty of Science
Branišovská 1760
CZ-37005 České Budějovice
petr.nguyen@prf.jcu.cz

Abstract

Genes for major ribosomal RNAs (rDNA) are present in multiple copies mainly organized in tandem arrays. Number and position of rDNA loci can change dynamically and their re-patterning is presumably driven by other repetitive sequences. We explored a peculiar rDNA organization in several representatives of Lepidoptera with either extremely large or numerous rDNA clusters. We combined molecular cytogenetics with analyses of second and third generation sequencing data to show that rDNA spreads as a transcription unit and reveal association between rDNA and various repeats. Furthermore, we performed comparative long read analyses among the species with derived rDNA distribution and moths with a single rDNA locus, which is considered ancestral. Our results suggest that satellite arrays, rather than mobile elements, facilitate homology-mediated spread of rDNA via either integration of extrachromosomal rDNA circles or ectopic recombination. The latter arguably better explains preferential spread of rDNA into terminal regions of lepidopteran chromosomes as efficiency of ectopic recombination depends on proximity of homologous sequences to telomeres.

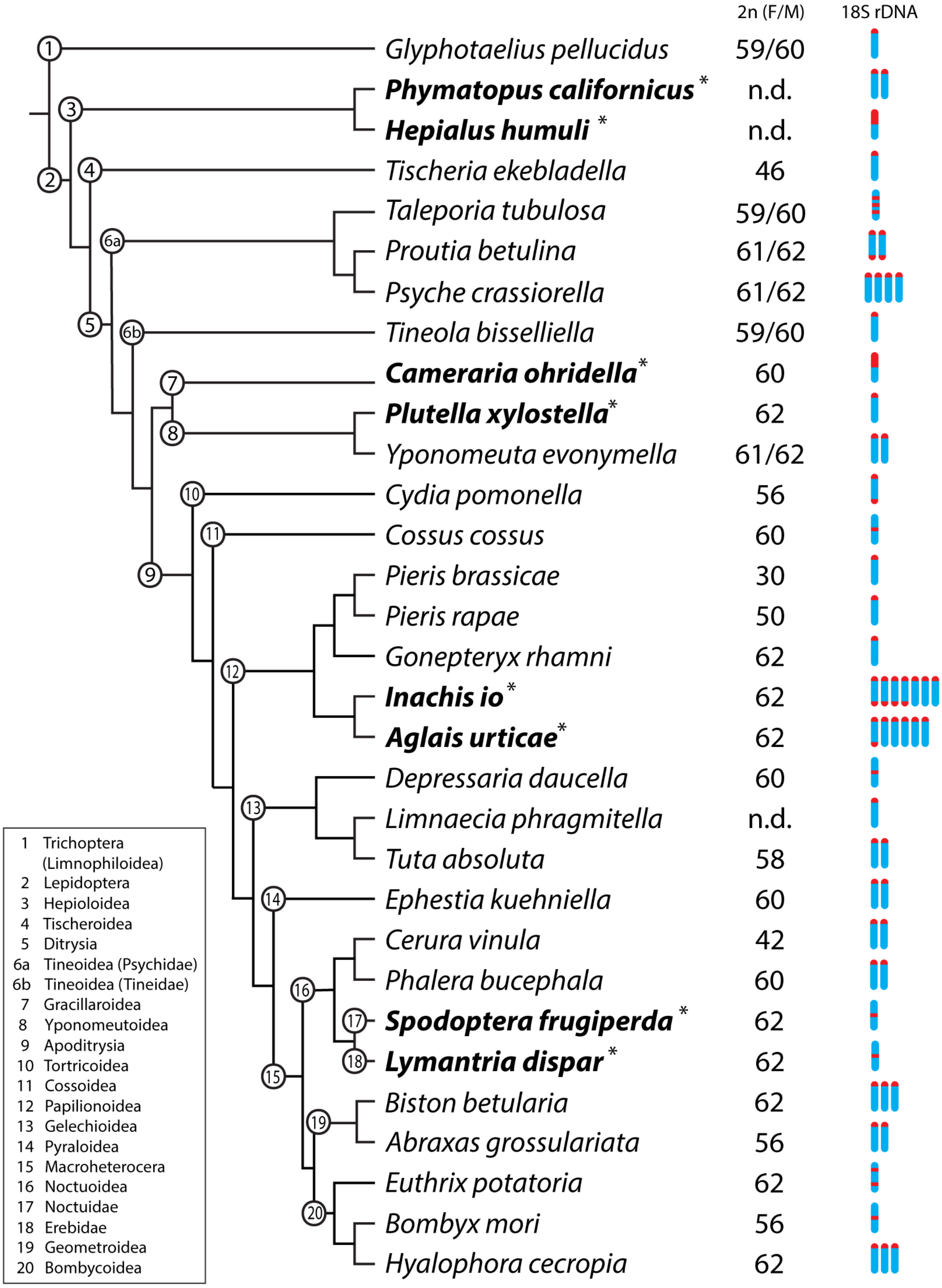

Figure S1: Overview of the number and position of 18S rDNA marker in haploid genomes of studied species adapted from Provazníková et al. (2021; doi:10.1038/s41598-021-91665-7) and references therein. Phylogenetic relationships are based on Kawahara et al. (2019; doi:10.1073/pnas.1907847116) and Wiemers et al. (2020; doi:10.3897/zookeys.938.50878). Note that Tineoidea are considered paraphyletic (6a, b). * Species included in this study (in bold). n.d.—not determined. F/M—female and male diploid chromosome numbers, if different. The figure was created in Adobe Illustrator 2020, version 24.0 (www.adobe.com).

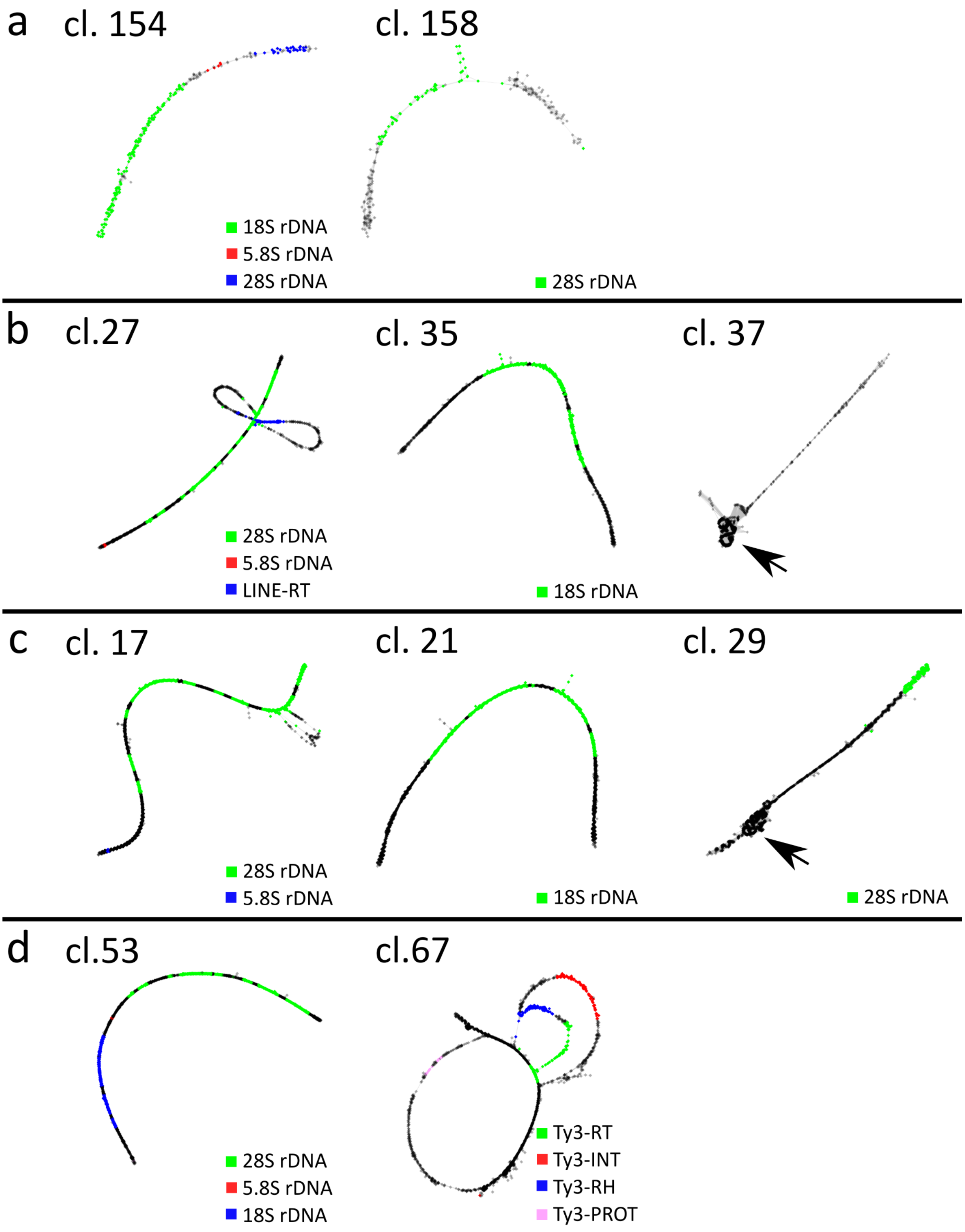

Figure S2: Graph layouts generated by the RepeatExplorer pipeline corresponding to clusters comprised in superclusters with detected rDNA genes. Nodes represent individual Illumina reads and edges their sequence overlap. **a**) *Cameraria ohridella*, **b**) *Inachis io*, **c**) *Aglais urticae*, and **d**) *Hepialus humulli*. Arrows indicate regions of graphs corresponding to satellite sequences as evidenced by their read density. rDNA genes and protein coding domains of mobile elements annotated by RepeatExplorer are colour coded. INT – integrase, RT – reverse transcriptase, PROT – protease, RH – Rnase H.

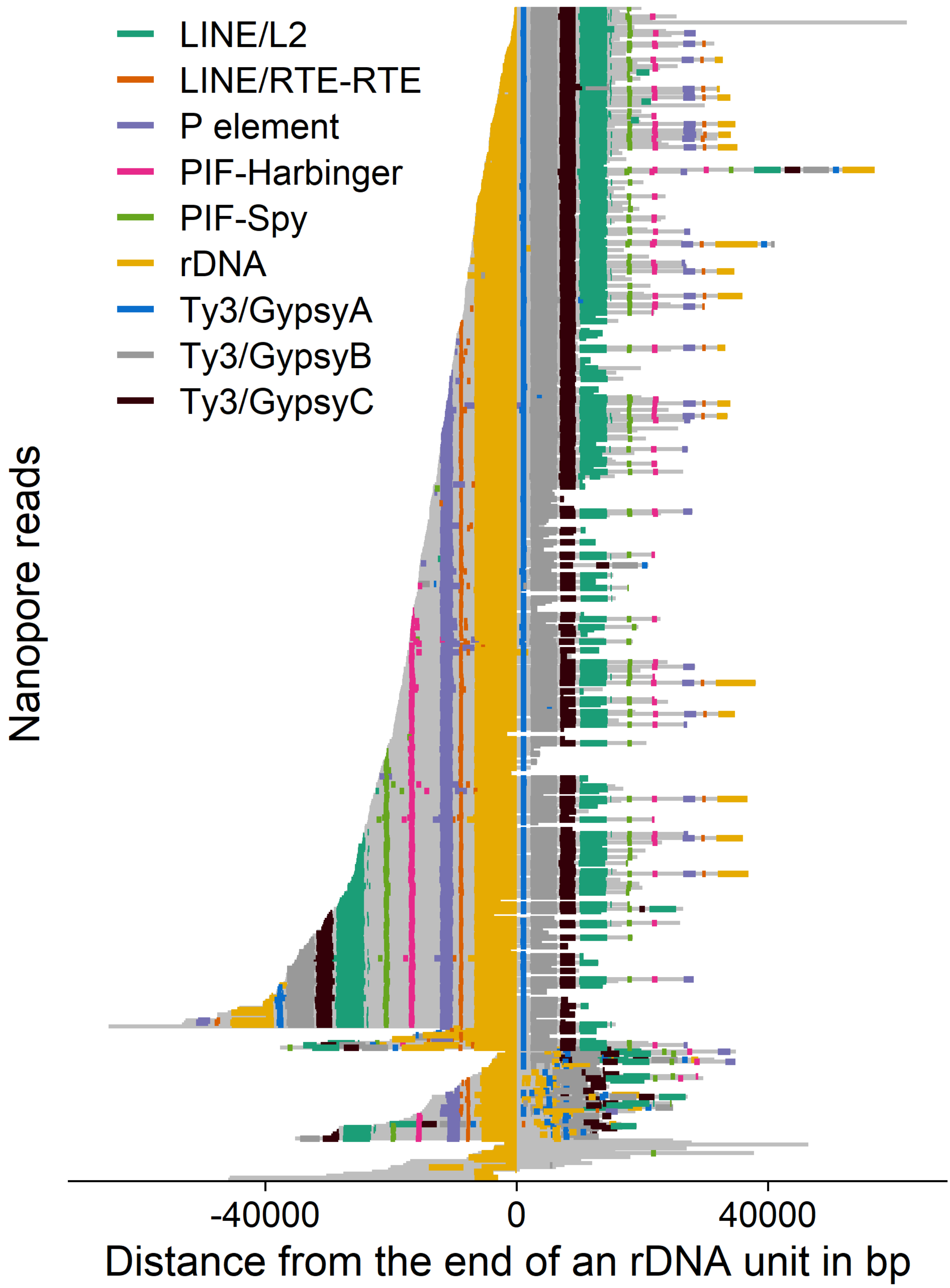

Figure S3: Visualization of major rDNA genes and associated repeats on *H. humuli* Oxford Nanopore reads. Only matches longer than 200 bp and with mapping quality over 20 were considered. Gray line represents the read length, coloured boxes correspond to the rDNA genes or individual detected repeats.

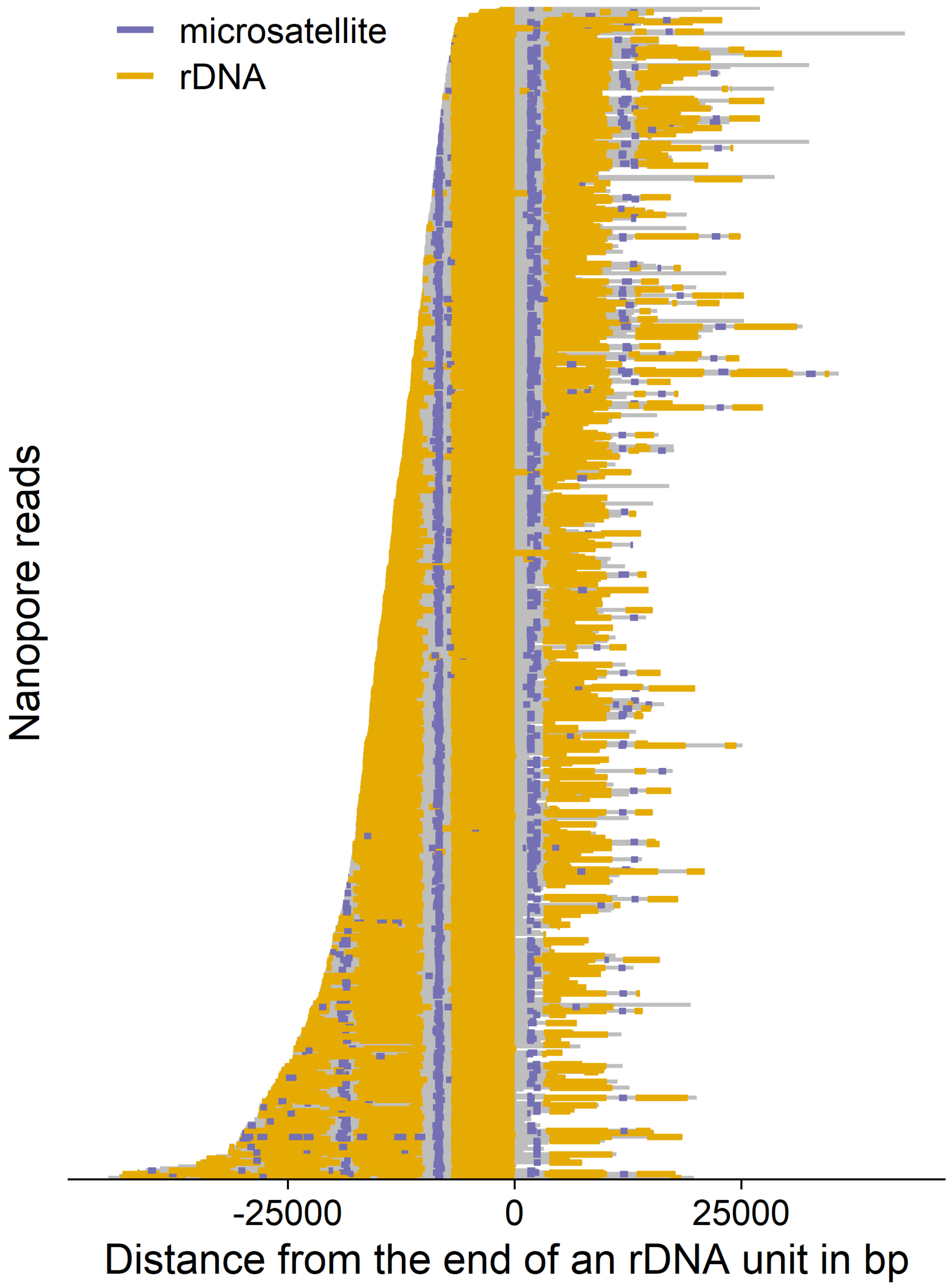

Figure S4: Visualization of major rDNA genes and associated repeats on *P. californicus* Oxford Nanopore reads. Only matches longer than 200 bp and with mapping quality over 20 were considered. Gray line represents the read length, coloured boxes correspond to the rDNA genes or individual detected repeats.

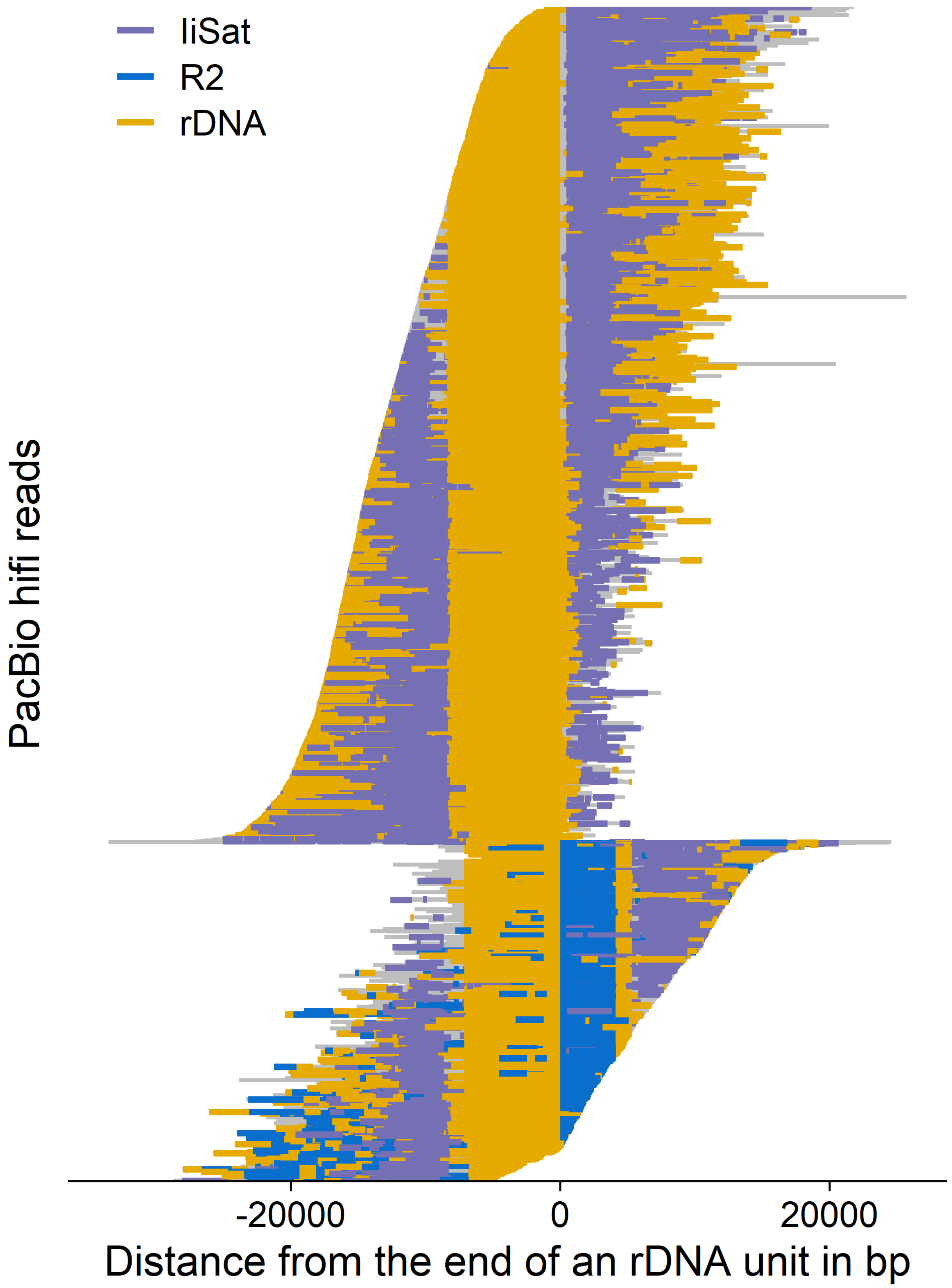

Figure S5: Visualization of major rDNA genes and associated repeats on *I. io* HiFi PacBio reads. Only matches longer than 200 bp and with mapping quality over 20 were considered. Gray line represents the read length, coloured boxes correspond to the rDNA genes or individual detected repeats.

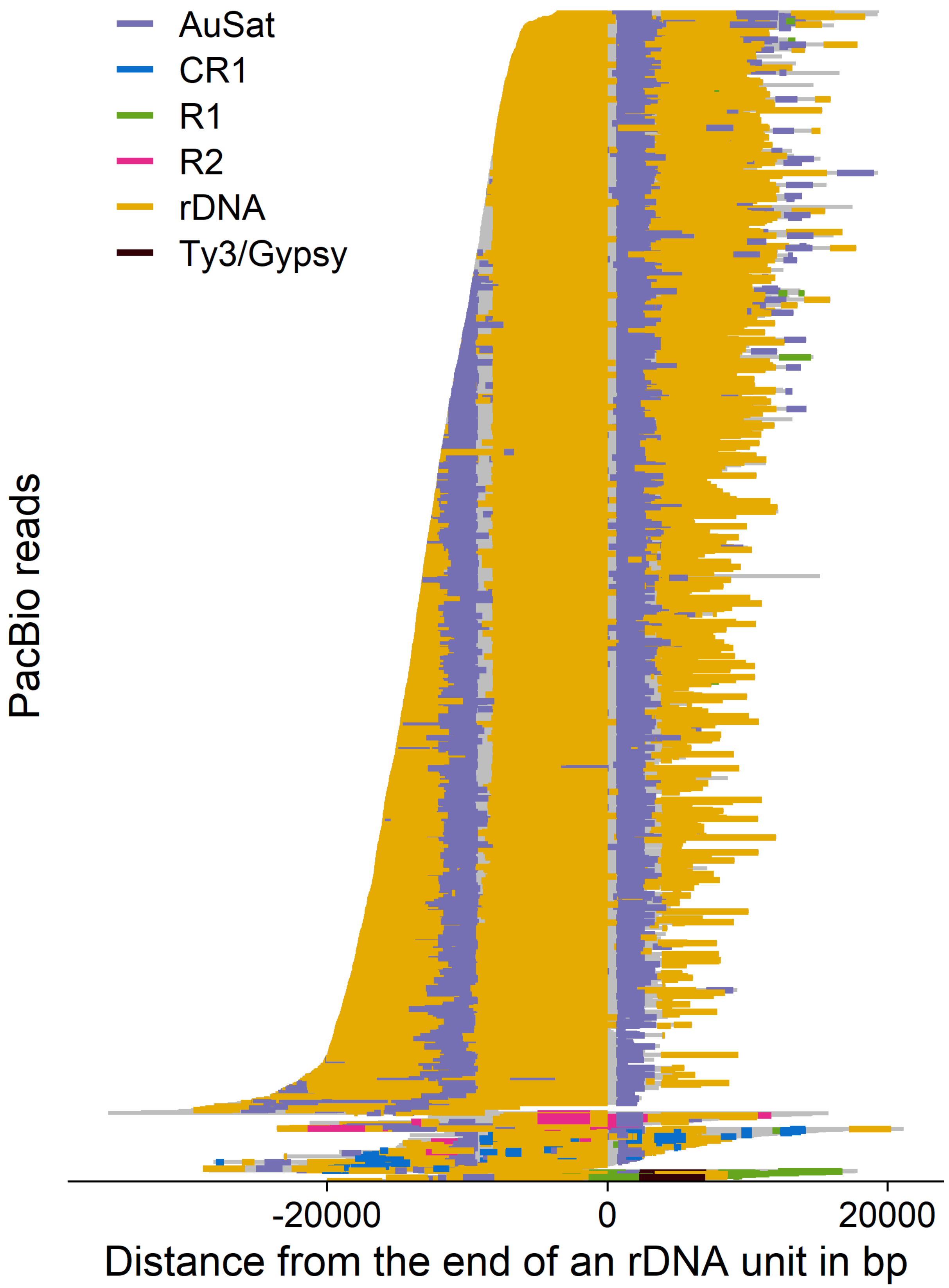

Figure S6: Visualization of major rDNA genes and associated repeats on *A. urticae* HiFi PacBio reads. Only matches longer than 200 bp and with mapping quality over 20 were considered. Gray line represents the read length, coloured boxes correspond to the rDNA genes or individual detected repeats.

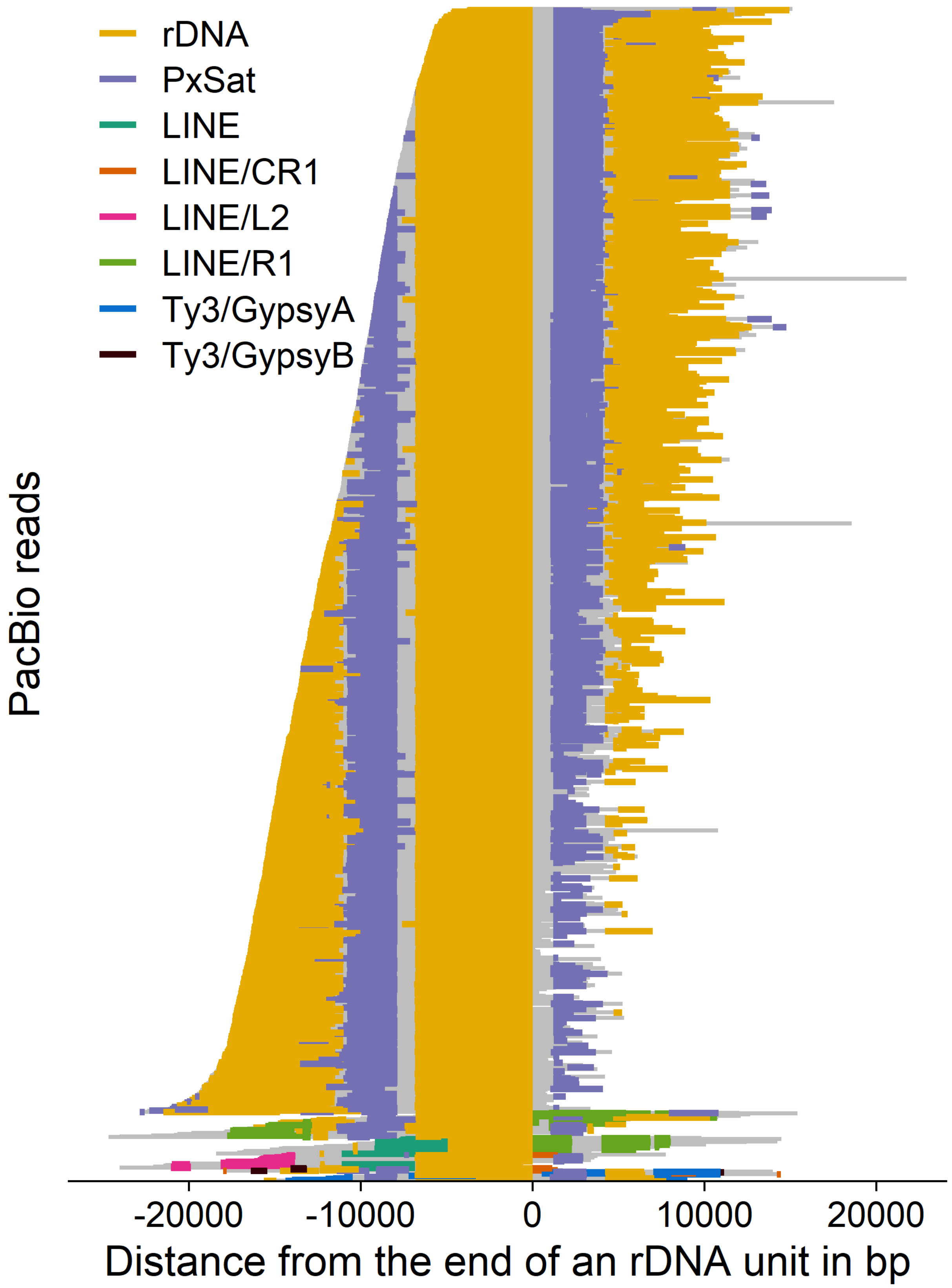

Figure S7: Visualization of major rDNA genes and associated satellite on *P. xylostella* PacBio reads. Only matches longer than 200 bp and with mapping quality over 20 were considered. Gray line represents the read length, coloured boxes correspond to the rDNA genes or PxSat.

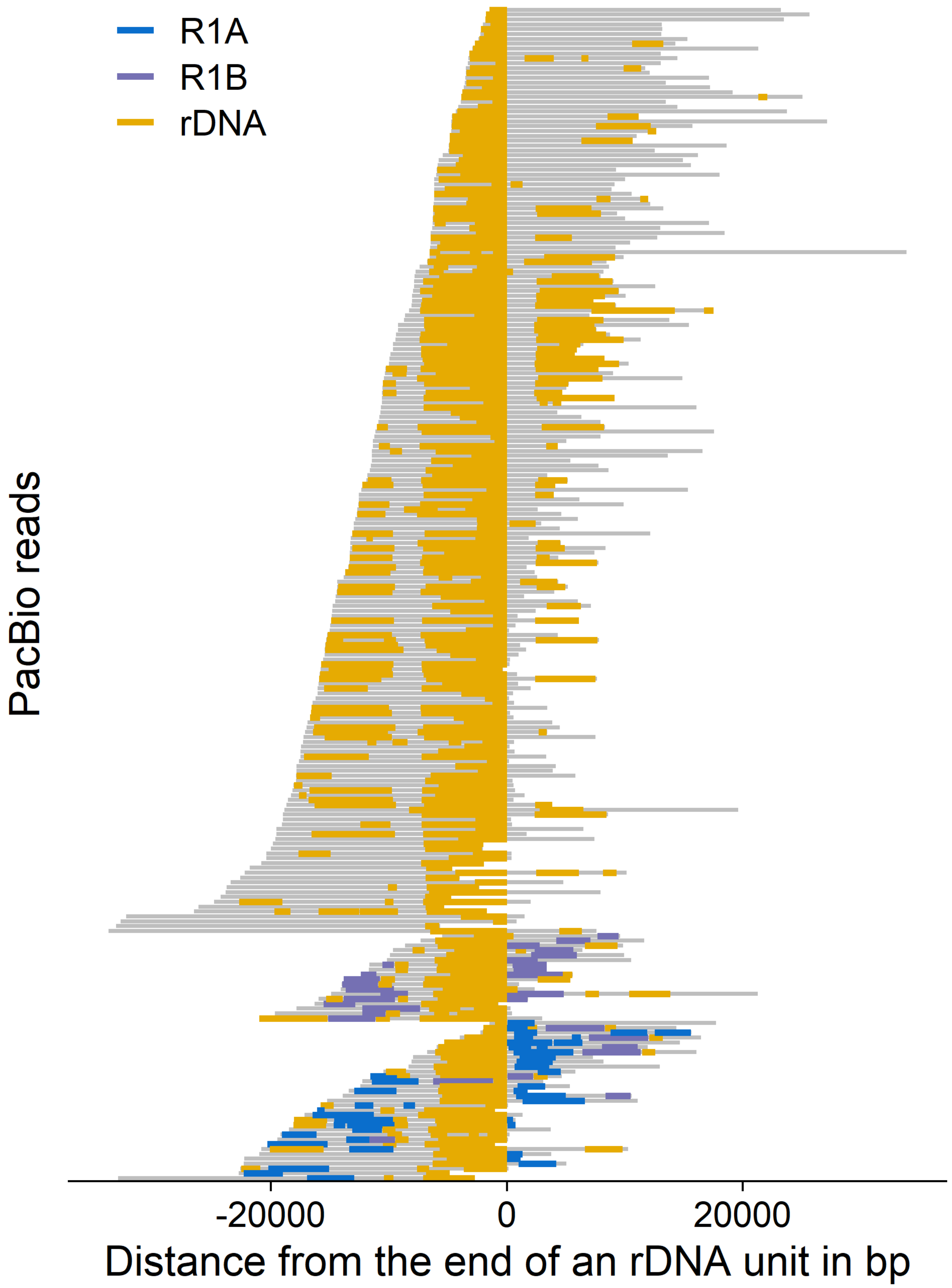

Figure S8: Visualization of major rDNA genes and associated repeats on *L. dispar* PacBio reads. Only matches longer than 200 bp and with mapping quality over 20 were considered. Gray line represents the read length, coloured boxes correspond to the rDNA genes or individual detected repeats.

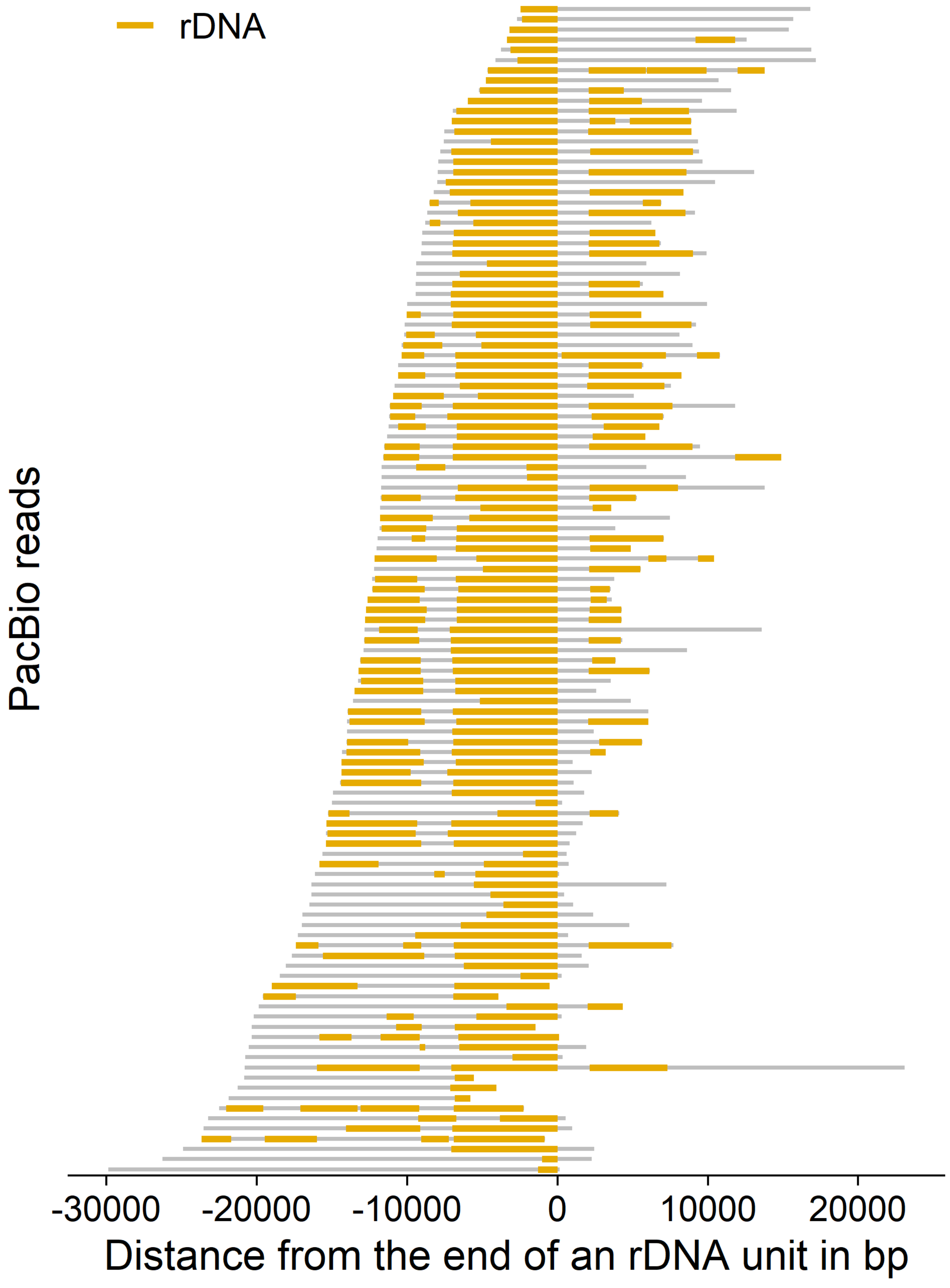

Figure S9: Visualization of major rDNA genes *S. frugiperda* PacBio reads. Only matches longer than 200 bp and with mapping quality over 20 were considered, no associated repeats were detected. Gray line represents the read length, coloured boxes correspond to the rDNA genes.

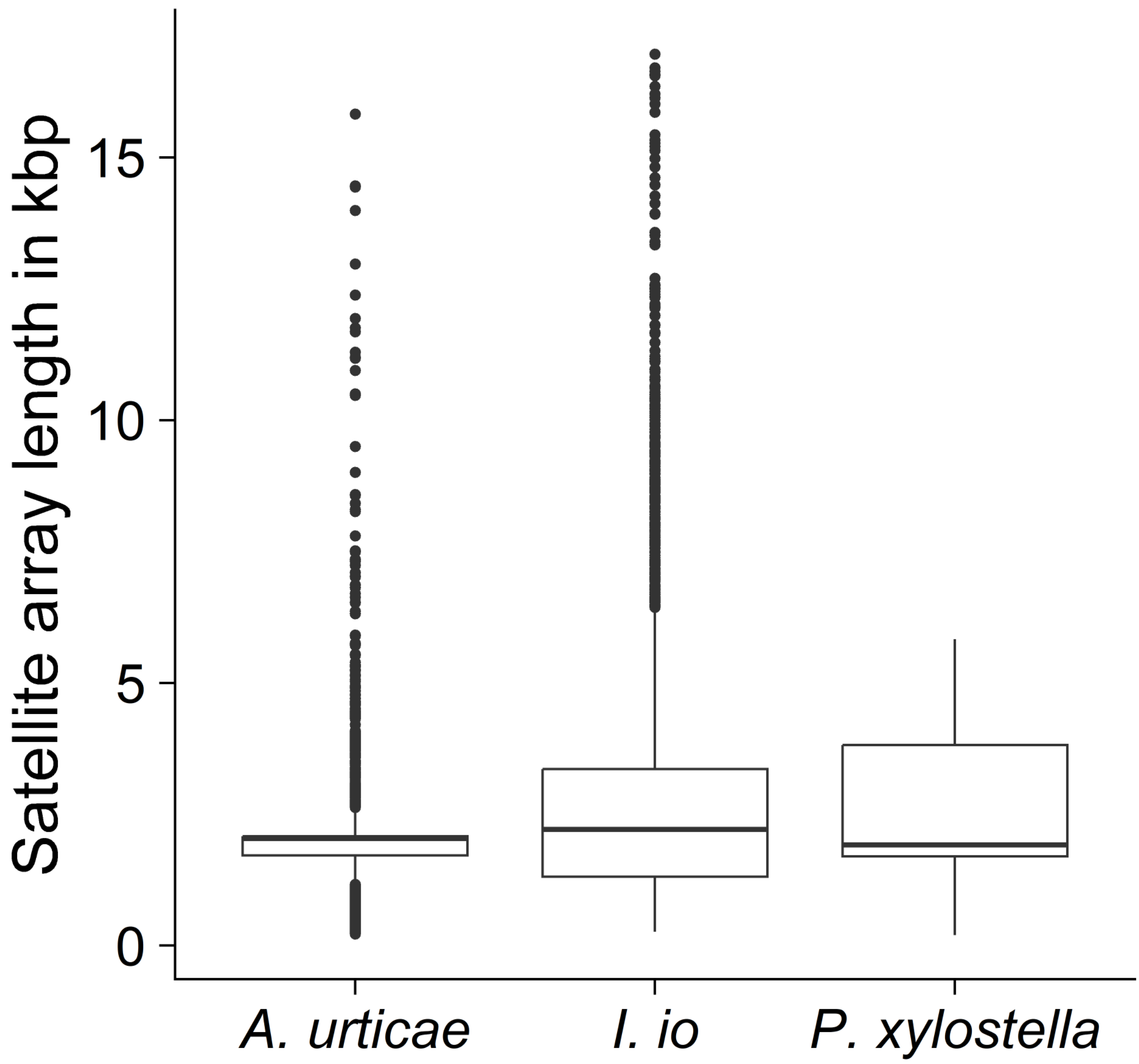

Figure S10: Distribution of AuSat (in *A. urticae*), IiSat (in *I. io*), and PxSat (in *P. xylostella*) arrays lengths in PacBio reads containing at least 500bp of major rDNA genes. Bold line represents median value, box corresponds to interquartile range, and the outlier values are represented by individual dots.

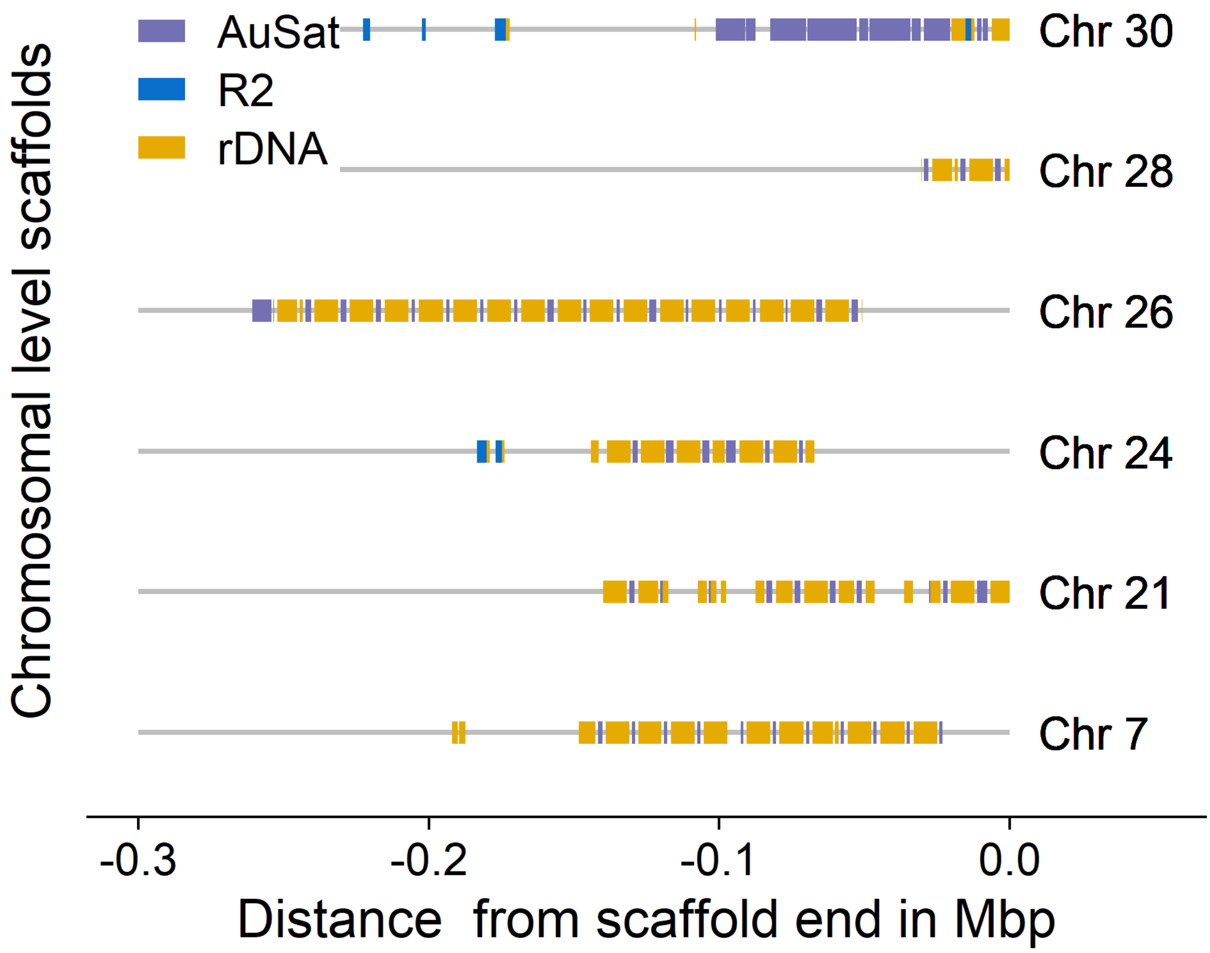

Figure S11: Visualization of major rDNA genes and associated repeats from the current *A. urticae* genome assembly. Only matches longer than 200 bp and with mapping quality over 20 were considered. Grey line represents the chromosomal length scaffolds with detected rDNA clusters, coloured boxes correspond to the rDNA genes or individual detected repeats.

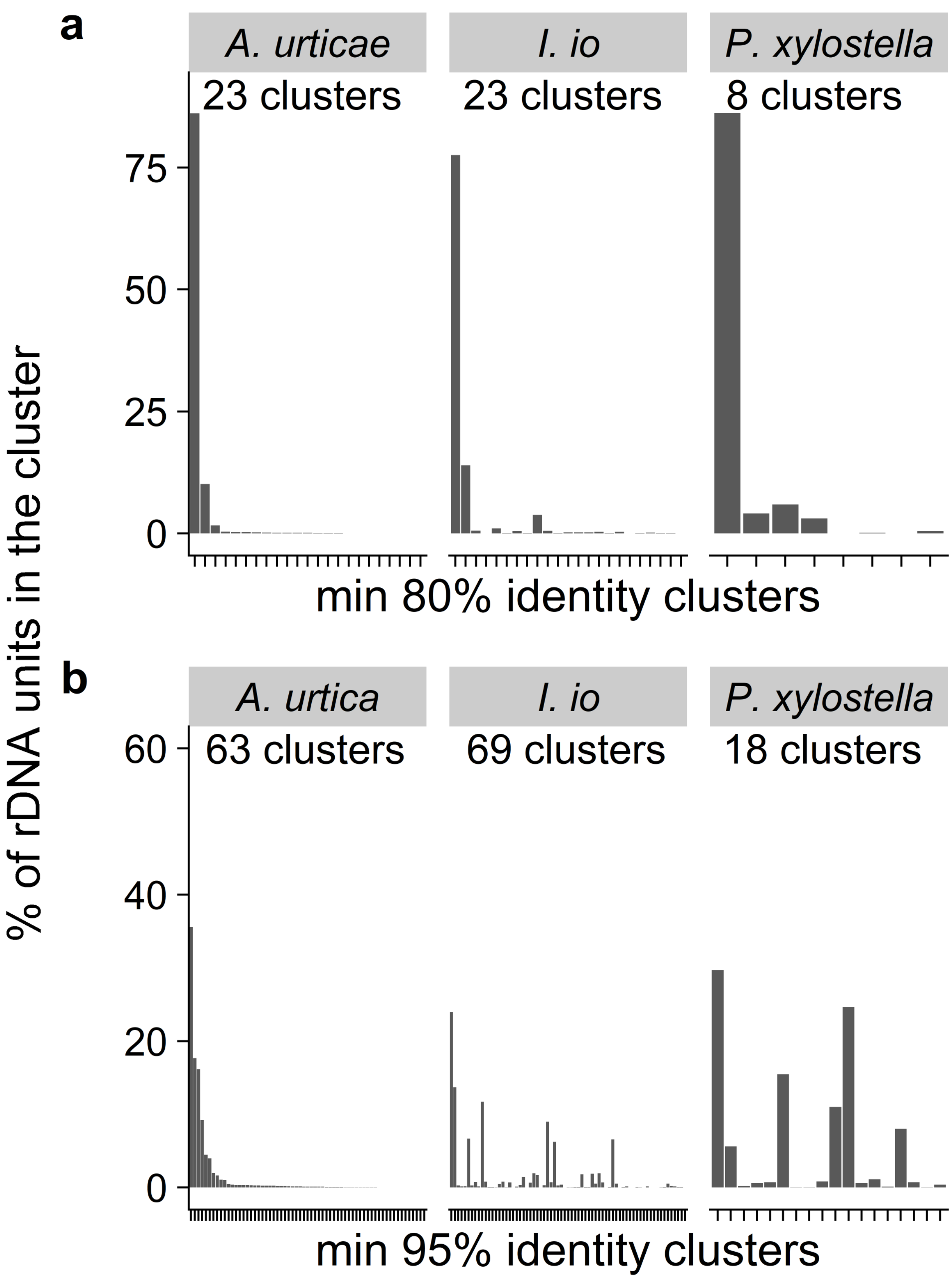

Figure S12: Visualization of clusters and percentage of rDNA units corresponding to individual clusters obtained by CD-HIT program analysis. The analysis was performed with two thresholds, 80% (**a**) and 95% (**b**) identity.
